# Geometric Constraints Regulate Energy Metabolism and Cellular Contractility in Vascular Smooth Muscle Cells by Coordinating Mitochondrial DNA Methylation

**DOI:** 10.1002/advs.202203995

**Published:** 2022-09-14

**Authors:** Han Liu, Yuefeng Liu, He Wang, Qiang Zhao, Tao Zhang, Si‐an Xie, Yueqi Liu, Yuanjun Tang, Qin Peng, Wei Pang, Weijuan Yao, Jing Zhou

**Affiliations:** ^1^ Department of Physiology and Pathophysiology School of Basic Medical Sciences; Hemorheology Center School of Basic Medical Sciences Peking University Beijing 100191 P. R. China; ^2^ Key Laboratory of Molecular Cardiovascular Science Ministry of Education Beijing 100191 P. R. China; ^3^ National Health Commission Key Laboratory of Cardiovascular Molecular Biology and Regulatory Peptides Beijing Key Laboratory of Cardiovascular Receptors Research Peking University Beijing 100191 P. R. China; ^4^ State Key Laboratory of Medicinal Chemical Biology Key Laboratory of Bioactive Materials Ministry of Education Collaborative Innovation Center of Chemical Science and Engineering (Tianjin) Nankai University Tianjin 300071 P. R. China; ^5^ Department of Vascular Surgery Peking University People's Hospital Beijing 100044 P. R. China; ^6^ Institute of Systems and Physical Biology Shenzhen Bay Laboratory Shenzhen 518132 P. R. China

**Keywords:** cPLA2, DNMT1, geometric constraint, mitochondrial DNA methylation, smooth muscle contractility

## Abstract

Vascular smooth muscle cells (SMCs) can adapt to changes in cellular geometric cues; however, the underlying mechanisms remain elusive. Using 2D micropatterned substrates to engineer cell geometry, it is found that in comparison with an elongated geometry, a square‐shaped geometry causes the nuclear‐to‐cytoplasmic redistribution of DNA methyltransferase 1 (DNMT1), hypermethylation of mitochondrial DNA (mtDNA), repression of mtDNA gene transcription, and impairment of mitochondrial function. Using irregularly arranged versus circumferentially aligned vascular grafts to control cell geometry in 3D growth, it is demonstrated that cell geometry, mtDNA methylation, and vessel contractility are closely related. DNMT1 redistribution is found to be dependent on the phosphoinositide 3‐kinase and protein kinase B (AKT) signaling pathways. Cell elongation activates cytosolic phospholipase A2, a nuclear mechanosensor that, when inhibited, hinders AKT phosphorylation, DNMT1 nuclear accumulation, and energy production. The findings of this study provide insights into the effects of cell geometry on SMC function and its potential implications in the optimization of vascular grafts.

## Graphical abstract

Two‐dimensional and three‐dimensional modeling of vascular smooth muscle cell geometry provides a platform for studying how cells respond to the physical stimuli. A critical role of the cPLA2‐AKT mechanotransduction cascade in sensing nuclear shape and modulating DNMT1 nuclear‐mitochondrial distribution is demonstrated. The findings indicate geometry a crucial determinant of cellular contractility and energy metabolism.

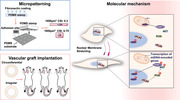

## 1 Introduction

Tissue architecture and organ morphogenesis are strongly influenced by cell geometry (shape, aspect ratio, and size), which is constrained and defined by the local tissue microenvironment organized by the cell–matrix and cell–cell interaction.^[^ ^1^ ^]^ Cell geometry plays a crucial role in determining cellular function.^[^ ^2^, ^3^ ^]^ Elongated cells must be equipped with arrangements that can guide the straightness of their long axis or with signal systems that can detect an undesired curvature. Symmetrically dividing cells must be able to locate their geometric centers accurately and determine the direction of the division plane according to their surface morphology.^[^ ^4^ ^]^ In the tunica media of the vasculature, vascular smooth muscle cells (SMCs) are responsible for maintaining vascular homeostasis through active contraction and relaxation and play an important role in vascular remodeling in health and disease.^[^ ^5^ ^]^ Vascular SMCs do not differentiate terminally, but retain a high plasticity, as they can be reprogrammed in response to vascular injury or alterations in local environmental cues, which leads to a phenotypic switch from quiescent/contractile to active/synthetic, accompanied by changes in the cellular geometry from an elongated spindle shape to a square or polygonal shape.^[^ ^6^ ^]^ Micro/nanosubstrate surface engineering is an established approach for modulating cellular behavior and function.^[^ ^7^ ^]^ Using the advantages of micropatterning technology, SMC elongation was shown to be tightly coupled with or cause the expression of smooth muscle contractile marker proteins, increase in cell contractility, repression of cell proliferation and migration, and alteration of cellular responses to growth factors and cytokines^[^ ^8^, ^9^, ^10^, ^11^, ^12^ ^]^; these findings suggest a crucial role of cell geometry‐conveyed mechanical cues in SMC functional regulation. However, despite intensive efforts to understand the effects of environmental stimuli on SMC function and their contribution to SMC‐related disease development, the mechanisms underlying SMC functional regulation in response to cell geometry are yet to be elucidated.

Changes in nuclear geometry have been found to correlate with changes in cell geometry.^[^ ^13^ ^]^ Mechanical forces exerted on cell surface mechanosensory proteins (such as integrins, ion channels, and G protein‐coupled receptors, among others)^[^ ^14^ ^]^ are also transmitted along the cytoskeletal filaments and concentrated in the cytoplasm and nucleus distant from the acting sites, to enable the action of the nucleus as a mechanotransducer.^[^ ^15^ ^]^ Emerging evidence indicates a direct role of the nucleus in mediating cellular mechanosensory and mechanotransduction cues.^[^ ^16^, ^17^ ^]^ The activation of cytosolic phospholipase A2 (cPLA2), a calcium‐dependent phospholipase, initiates the release of arachidonic acid, which modulates inflammatory reactions and regulates lipid metabolism. Recent studies based on controlled three‐dimensional microconfinement and osmotic shock experiments have shown that cPLA2 is a mechanosensitive protein that can be activated by the deformation of the nuclear membrane.^[^ ^18^, ^19^ ^]^ The nucleus senses changes in cellular shape in the form of cell/nuclear swelling and stretching and consequently triggers the translocation of cPLA2 from its resting localization in the nucleoplasm to the nuclear membrane.^[^ ^18^, ^19^ ^]^ However, despite its role in regulating cellular inflammation and lipid metabolism, the activation of cPLA2 and the subsequent release of arachidonic acid may lead to phenotypic switching and cell proliferation via the activation of the phosphoinositide 3‐kinase (PI3K) and protein kinase B (AKT) signaling pathways in various types of cells.^[^ ^20^, ^21^ ^]^ In vascular SMCs, the cPLA2‐arachidonic acid‐PI3K‐AKT pathway mediates cell proliferation triggered in response to biochemical stimuli (e.g., angiotensin II, adenosine triphosphate (ATP), and insulin.^[^ ^22^, ^23^, ^24^ ^]^ Nevertheless, whether this signaling cascade plays a role in transducing mechanical forces mediated via cell geometry in vascular SMCs remains elusive.

Mitochondrial DNA (mtDNA) is a double‐stranded, circular molecule of 16569 bp containing 37 genes encoding 13 proteins, 22 tRNAs, and 2 rRNAs. The 13 mtDNA‐encoded proteins constitute the enzyme complexes of the oxidative phosphorylation system, which enable the mitochondria to function as the powerhouse of cells.^[^ ^25^ ^]^ Mutation in or the insufficient expression of mitochondrial genes has been observed in various diseases, such as cancer, diabetic retinopathy, multiple sclerosis, and atherosclerosis.^[^ ^26^, ^27^, ^28^, ^29^ ^]^ The abrogation of mitochondrial transcription by the depletion of mitochondrial transcription factor A (TFAM) in vascular SMCs impairs smooth muscle contractility and vascular tone.^[^ ^30^ ^]^ MtDNA contains a unique 1124 bp noncoding region known as the displacement loop (D‐loop), which controls mtDNA replication and promotes mitochondrial gene transcription.^[^ ^31^ ^]^ Mitochondrial D‐loop methylation has been observed in cells of various types, including vascular SMCs, and has been suggested to be associated with the transcriptional repression of mtDNA genes.^[^ ^29^, ^32^ ^]^ DNA methyltransferase 1, ‐3A, and ‐3B (DNMT1, DNMT3A, and DNMT3B, respectively), which are present in the mitochondria, have been attributed in the catalysis of D‐loop methylation at cytosine‐phospho‐guanine (CpG) dinucleotides.^[^ ^33^, ^34^, ^35^ ^]^ We reported that in vascular SMCs, DNMT1 undergoes nuclear‐to‐mitochondrial redistribution upon stimulation with platelet‐derived growth factor‐BB (PDGF‐BB).^[^ ^29^ ^]^ Mitochondria‐localized DNMT1 can bind to the D‐loop and may eventually cause the repression of mtDNA gene transcription, impairment of mitochondrial oxidative phosphorylation, and loss of smooth muscle contractility.^[^ ^29^ ^]^ We speculate that DNMT1 redistribution may be triggered by changes in cell geometry as well, and the mechanism underlying DNMT1 translocation by cell elongation or shortening could help elucidate novel mechanotransduction pathways.

In this study, we showed that geometric stimuli trigger a series of intrinsically connected effects in vascular SMCs, including the subcellular redistribution of DNMT1, changes in the mtDNA methylation levels, binding of DNMT1 to the D‐loop, expression of mtDNA genes, and mitochondrial function, along with alterations in energy metabolism. We demonstrated the role of the nuclear mechanosensory protein cPLA2 and its downstream signaling molecules PI3K and AKT1 in mediating these mechanical force‐elicited cellular effects. Our results indicate that cell geometry is a crucial determinant of cell function and reveals a previously unidentified mechanism in the phenotypic modulation of vascular SMCs.

## 2 Results

### 2.1 Vascular SMCs Exhibit Distinct Cell Geometry in Normal and Diseased Vasculature

Using transmission electron microscopy, irregular polygon‐shaped SMCs were detected in diseased vasculature (atherosclerotic plaques from carotid endarterectomy and the tunica media obtained during surgical treatment for aortic dissection) (Figure S1, Supporting Information). The cell shape indexes (CSIs) were ≈0.7689 and 0.5357, respectively. Notably, the cells present in the plaque areas had a long axis that was shorter than that of cells in the plaque‐adjacent areas. In contrast, cells in the normal internal mammary artery were mostly elongated, with a mean CSI of 0.3295. In an experimental mouse model for the investigation of vascular SMC activation, complete ligation of the carotid artery induced neointima formation (Figure S2A, Supporting Information), largely owing to SMC proliferation and migration.^[^ ^36^ ^]^ Immunofluorescent staining of the smooth muscle marker transgelin/SM22*α*, followed by the quantification of cell shape, indicated a significant difference between the CSIs of cells in the unligated tunica media (0.4394) and ligated media/neointima (0.6389/0.7429), but no significant difference between the CSIs of cells in the ligated tunica media and neointima (Figure S2B, Supporting Information). These observations suggest that SMC shape may be indicative of vascular health or disease.

### 2.2 Geometric Constraints Induce the Redistribution of DNMT1

**Figure 1:**
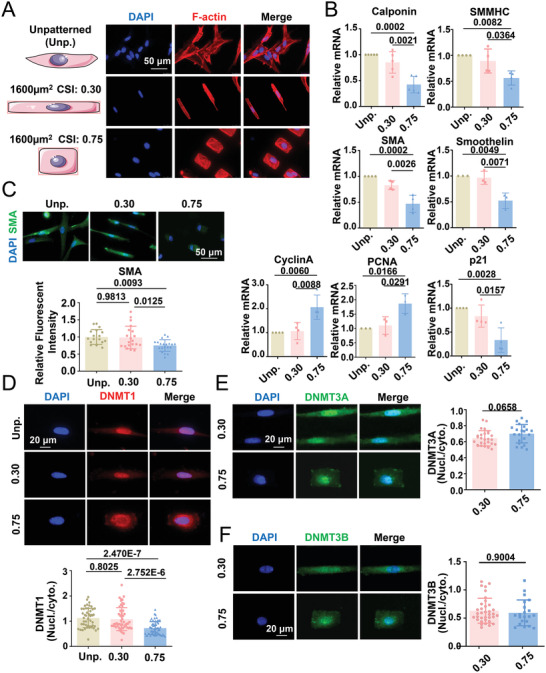
Geometric constraints induce changes in vascular smooth muscle phenotype and the subcellular distribution of DNA methyltransferase (DNMT) 1. A) Representative fluorescent staining of F‐actin in unpatterned (Unp.) and micropatterned (CSI = 0.30 and CSI = 0.75) smooth muscle cells (SMCs). B) The mRNA levels of genes determining the smooth muscle phenotype were measured using quantitative RT‐PCR. Data were obtained from three to five biological repeats. C) Representative immunofluorescence staining and quantification of the expression of the smooth muscle contractile gene, smooth muscle alpha‐actin (SMA). D–F) Representative immunofluorescence staining and quantification of the nuclear/cytoplasmic ratio of DNMT1, DNMT3A, and DNMT3B. In (D)–(F), each dot represents a single cell. Fluorescence images were acquired using an epifluorescence microscope. Significance was assessed using E,F) a Student's *t*‐test and B–D) one‐way ANOVA with Tukey's post hoc analysis. The error bars show ± SD. The exact *P* values between the indicated groups are presented.

To manipulate the cell geometry, human umbilical artery SMCs were seeded on elastic polydimethylsiloxane substrates (PDMS) with fibronectin micropatterns or un‐patterns. The micropatterns were either elongated (CSI = 0.30) or square‐shaped (CSI = 0.75), with an equal area (1600 µm^2^) (Figure S3, Supporting Information and **Figure** **1**A). F‐actin staining showed that cells that spread on the micropatterned substrates were restricted to the engineered geometry (Figure 1A). Analysis of the expression of smooth muscle contractile markers, such as calponin, smooth muscle myosin heavy chain, smooth muscle *α*‐actin, and smoothelin, cell proliferation markers cyclin A and proliferating cell nuclear antigen, and the cell cycle inhibitor p21, led to the identification of a pro‐contractile phenotype in the elongated cells and a pro‐synthetic phenotype in the square‐shaped cells (Figure 1B,C). The unpatterned cells resembled elongated cells with respect to the gene expression characteristics (Figure 1B). These results are in line with previous findings^[^ ^8^, ^12^ ^]^ and indicate the coordination of cell behavior and geometry. To visualize the response of DNMT1 to geometric restriction, unpatterned or micropatterned cells were stained for DNMT1 (antibody specificity is validated in Figure S4, Supporting Information) at 24 h after seeding. Intriguingly, nuclear DNMT1 expression was prominent in both unpatterned and elongated‐patterned cells, but DNMT1 exhibited nuclear and cytoplasmic localization in the square‐shape‐patterned cells (Figure 1D). In SMCs residing in atherosclerotic plaques, DNMT1 also showed cytoplasmic accumulation (Figure S5, Supporting Information). Treatment of the cells with leptomycin B, which blocks chromosomal region maintenance 1‐dependent nuclear export, promoted the nuclear retention of DNMT1 in the micropatterned cells, which confirmed the regulation of DNMT1 distribution (Figure S6, Supporting Information). Quantification of the nuclear/cytoplasmic DNMT1 distribution ratio revealed significant differences between the cells with CSI 0.30 and 0.75 and between unpatterned cells and cells with CSI 0.75, but no significant difference between unpatterned cells and cells with CSI 0.30 (Figure 1D). In addition, SMCs on the unpatterned substrate showed an elongated morphology (Figure S7, Supporting Information). Thus, in subsequent experiments, comparisons were made only using micropatterned cells.

The differential regulation of the subcellular distribution of molecules in response to geometric constraints did not apply to DNMT3A or DNMT3B, as their localization was unaltered in response to cell elongation or shortening (Figure 1E,F). The application of the constraints of growth space with different cell spreading areas (800, 1200, or 1600 µm^2^) did not significantly alter the DNMT1 nuclear/cytoplasmic ratio in cells with a fixed CSI (Figure S8, Supporting Information), which suggests the importance of shape, despite changes in the cell growth space.

### 2.3 Geometric Constraints Regulate mtDNA Methylation

**Figure 2:**
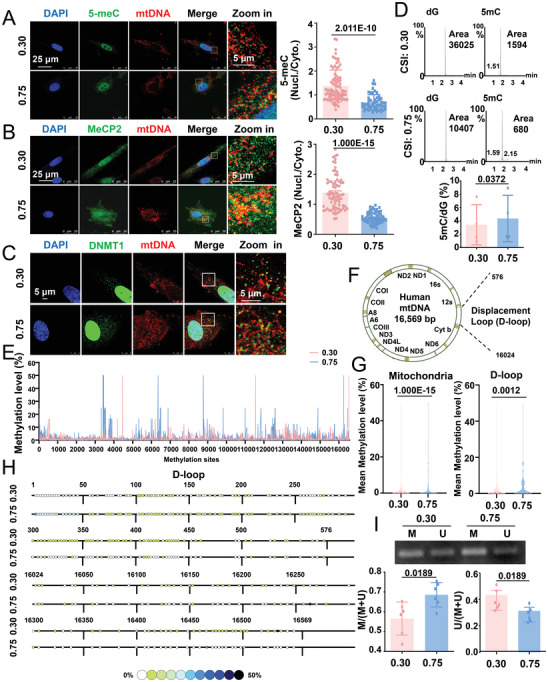
Geometric constraints regulate the methylation of mitochondrial DNA (mtDNA). A,B) Representative immunofluorescence staining and quantification of the nuclear/cytoplasmic ratio of 5‐methylcytosine (5‐meC) and MeCP2 in micropatterned SMCs. MtDNA was co‐stained with an anti‐mtDNA antibody. Each dot represents a single cell. C) Representative immunofluorescence staining of DNMT1 and mtDNA in micropatterned cells. D) Representative multiple reaction monitoring chromatograms of 5‐methyl‐deoxy‐cytidine (5‐mdC) and deoxyguanosine (dG) in the mtDNA of micropatterned cells. Quantitative statistical analysis of 5‐meC in micropatterned cells. Data were obtained from four biological repeats. E) Methylation patterns of the mtDNA of micropatterned cells. Schematic representation of the cytosine residue. Data were obtained from two duplicate samples. Each sample was obtained from four biological repeats. F) Schematic diagram of the human mitochondrial genome and D‐loop regions. G) The mean 5‐meC level of the entire mtDNA (left panel) and D‐loop (right panel) in micropatterned cells. H) Distribution of the D‐loop 5‐meC content in micropatterned cells. I) Representative agarose gel electrophoresis of the products of methylation‐specific PCR (MSP) for the D‐loop in bisulfite‐modified DNA isolated from the micropatterned cells. Semi‐quantification of the MSP results is shown in the lower panels. M: methylated, U: unmethylated. Immunofluorescent images were acquired using a confocal microscope. Significance was assessed using A,B) an unpaired *t*‐test, D,I) paired *t*‐test, or G) the Mann‐Whitney test. The error bars show ± SD. The exact *P* values between the indicated groups are presented.

Findings from studies conducted by us and other research groups have suggested the presence of DNMT1 and 5‐methylcytosine (5‐meC) within the mitochondria in vascular SMCs and other types of cells.^[^ ^29^, ^37^, ^38^ ^]^ Immunofluorescent staining results indicated that the nuclear/cytoplasmic ratio of 5‐meC was lower but 5‐meC and mtDNA colocalization was higher in square‐shaped cells than in the elongated cells (**Figure** **2**A), which suggested the influence of cell shape in the regulation of nonnuclear DNA methylation. Methyl‐CpG‐binding protein 2 (MeCP2) is a global transcription factor that forms a complex with DNMT1 to bind methylated DNA.^[^ ^39^ ^]^ The prominent cytoplasmic retention of MeCP2 was observed in squared‐shaped cells, and the increase in the CSI from 0.30 to 0.75 significantly affected the nuclear/cytoplasmic ratio of MeCP2 (Figure 2B), which is in line with the observations for DNMT1. The results of immunofluorescence staining showed that the colocalization of DNMT1 with mtDNA was more pronounced in square‐shaped cells (Figure 2C). Stimulated emission depletion (STED) super‐resolution microscope imaging confirmed a stronger colocalization of DNMT1 in the mitochondria of square‐shaped cells, as an increase in the CSI from 0.30 to 0.75 induced an increase of 30.6% in the correlation coefficient between DNMT1 and the mitochondrial membrane protein ATP synthase, H^+^‐transporting, mitochondrial F1 complex, alpha subunit 1 (Figure S9, Supporting Information).

To directly investigate the effect of cell geometry on mtDNA methylation, mtDNA was isolated from micropatterned cells, purified, digested, and subjected to high‐performance liquid chromatography‐mass spectrometry (HPLC‐MS) for the quantification of mtDNA methylation. MtDNA was amplified using PCR, followed by gel electrophoresis, and the absence of genomic DNA‐encoded genes and presence of mtDNA‐encoded genes was confirmed to verify the purity of the sample (Figure S10, Supporting Information). With an increase in the CSI, the ratio of 5‐methyl‐deoxy‐cytidine to deoxyguanosine increased from 3.425% ± 1.511% to 4.354% ± 1.744% (Figure 2D), which indicated higher mtDNA methylation levels in square‐shaped cells. To confirm geometry‐regulated mtDNA methylation, mtDNA from micropatterned cells was subjected to bisulfite sequencing (Figure 2E). Two hundred and twelve methyl‐cytosine sites were identified in the D‐loop region, of which 137 and 104 methylated sites (methylation level > 0%) were identified in the elongated versus square‐shaped cells, respectively (Figure 2F–H). The methylcytosine levels in the mitochondria were 1.465% (min = 0 and max = 50%) in the elongated cells and 3.106% (min = 0, max = 50%) in the square‐shaped cells, whereas those in the D‐loop region were 1.085% (min = 0 and max = 5%) in the elongated cells and 2.111% (min = 0, max = 50%) in the square‐shaped cells (Figure 2G). The methylation status of the mitochondrial D‐loop was further evaluated by methylation‐specific PCR (MSP) using primers targeting the D‐loop region, which helped confirm hypermethylation in square‐shaped cells (Figure 2I). Collectively, these data indicate that the subcellular distribution of DNMT1, DNMT1‐associated MeCP2, and 5‐meC and the methylation of mtDNA are influenced by cell geometry.

### 2.4 The binding of DNMT1 to mtDNA and the transcription of mtDNA‐encoded genes are regulated by cell geometry

**Figure 3:**
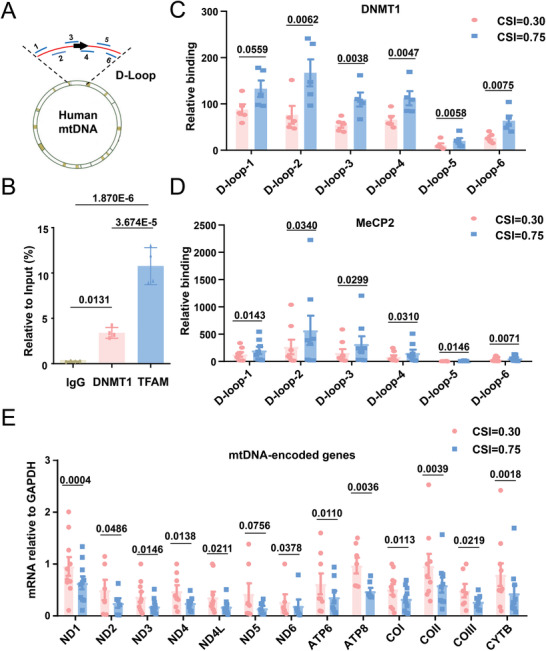
DNMT1 binding to mtDNA and the transcription of mtDNA‐encoded genes are regulated by geometric constraints. A) Schematic representation of the human mitochondrial genome with the indication of the locations of the primer sets for the chromatin immunoprecipitation (ChIP) assay. B) ChIP assay to determine the binding of DNMT1 to the D‐loop regions in purified mitochondria from unpatterned SMCs. Transcription factor A, mitochondrial (TFAM) served as a positive control. Data were obtained from four biological repeats. C,D) ChIP assay to determine the binding of DNMT1 and MeCP2 to the D‐loop regions in micropatterned cells. Data were obtained from six biological repeats. E) The mRNA levels of mtDNA‐encoded genes were determined using quantitative RT‐PCR. Data were obtained from ten biological repeats. Significance was assessed using B) one‐way ANOVA with Tukey's post hoc analysis or C–E) a Student's *t*‐test. The error bars show ± SD. The exact *P* values between the indicated groups are presented.

The cell geometry‐regulated binding of DNMT1 to mtDNA was postulated to yield functional outcomes. To prove this, we performed a chromatin immunoprecipitation assay to detect the direct binding of DNMT1 to the mtDNA D‐loop region using six overlapping PCR primer sets spanning the 1 to 604 regions of human mtDNA (**Figure** **3**A). The binding specificity was first tested in purified mitochondria from unpatterned SMCs, using negative (IgG) and positive (TFAM) controls (Figure 3B). In micropatterned SMCs, binding detected by five of the six tested primer sets was significantly promoted with an increase in the CSI, which suggested a stronger association of DNMT1 with mtDNA in the square‐shaped cells (Figure S11, Supporting Information and Figure 3C). Consistently, the binding of MeCP2 to the D‐loop underwent a marked increase when the CSI increased from 0.30 to 0.75 (Figure 3D). Next, the transcription of 13 genes encoded by mtDNA was analyzed in the micropatterned cells. Most of the genes (12 out of 13) showed significant differences in expression between the elongated and square‐shaped cells and notable repression in the square‐shaped cells (Figure 3E).

### 2.5 Cell Geometry Affects Mitochondrial Function and Energy Metabolism

**Figure 4:**
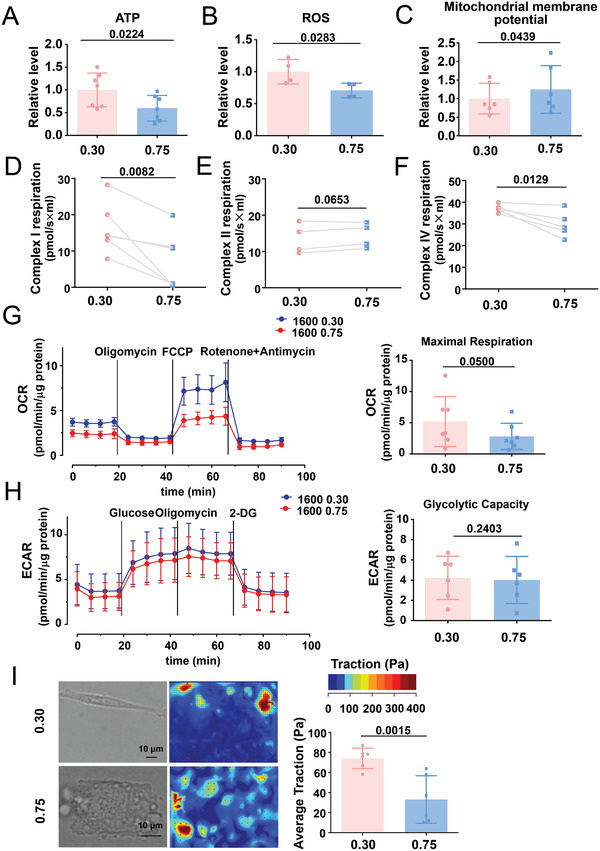
Cells with different geometry differ in mitochondrial function and energy metabolism. A) Intracellular adenosine triphosphate (ATP) content in the micropatterned SMCs was evaluated using a photometer. Data were obtained from seven biological repeats. B) The production of reactive oxygen species in micropatterned cells was assayed using flow cytometry. Data were obtained from four biological repeats. C) The mitochondrial membrane potential was measured using flow cytometry. Data were obtained from six biological repeats. D–F) Complex I‐, II‐, and IV‐supported respiration was analyzed by Oroboros Oxygraph‐2k, a high‐resolution respiration analyzer. Data were obtained from five biological repeats. G) Representative traces of the oxygen consumption rate (OCR) measured using the Seahorse XF96 flux analyzer. Mitochondrial effectors were injected sequentially at the time points indicated by the arrows, and the maximum OCR was analyzed. Data were obtained from seven biological repeats. H) Representative traces of the extracellular acidification rate (ECAR) measured using the Seahorse XF96 flux analyzer. Mitochondrial effectors were sequentially injected at the time points indicated by the arrows. The glycolytic capacity was analyzed. Data were obtained from seven biological repeats. I) Traction force microscopy assay to detect cellular contractility in micropatterned SMCs. Representative bright field images and cell force images are presented in the left panel. Quantification of the traction is shown in the right panel. Each dot represents a single cell. Significance was assessed using A–C,G–I) the Student's *t*‐test or D–F) paired *t*‐test. The error bars show ± SD. The exact *P* values between the indicated groups are presented.

We explored the cellular functional outcomes of the geometric constraints. SMC contraction requires the consumption of ATP, and mtDNA genes encode proteins for the assembly and activity of mitochondrial respiratory complexes.^[^ ^40^ ^]^ Compared with the elongated cells, the square‐shaped cells showed lower intracellular levels of ATP (**Figure** **4**A), lower reactive oxygen species (ROS) production (Figure 4B), elevated mitochondrial membrane potential (Figure 4C), and enhanced mitochondrial fission with inhibition of fusion (Figure S12, Supporting Information), which indicated the impairment of mitochondrial function. The number of autophagosomes (indicated by LC3) with overlapping staining patterns of the mitochondrial outer membrane marker TOM20 indicated no apparent mitophagy activation in the micropatterned SMCs (Figure S13, Supporting Information). The O2k mitochondrial function assay system was used to detect the activity of the mitochondrial respiratory complexes. Respiratory complex I‐ and IV‐supported respiratory function was markedly reduced in the square‐shaped cells, whereas respiratory complex II‐supported respiratory function showed no significant change (Figure 4D–F), probably because the mtDNA‐encoded proteins are components of mitochondrial respiratory complexes I and IV, but not complex II.^[^ ^25^ ^]^ Furthermore, we used the Seahorse extracellular flux analyzer to determine the levels of oxidative phosphorylation (indicated by the oxygen consumption rate, OCR) and glycolysis (indicated by the extracellular acidification rate) in the micropatterned cells. Compared with the elongated cells, the square‐shaped cells exhibited a considerably lower maximum OCR and a comparable glycolytic capacity (Figure 4G,H), which indicated the differential regulation of oxidative phosphorylation by cell geometry. To directly link cell geometry to cellular energy metabolism powered by mitochondrial oxidative phosphorylation, we analyzed the consequent cellular contractility at the single‐cell level using traction force microscopy in micropatterned SMCs. Cells with an elongated shape were equipped with higher traction forces than those with a square shape, which suggests a strong association between cell geometry and contractility (Figure 4I). These results support the notion that cell geometry affects energy metabolism in vascular SMCs.

### 2.6 SMC Geometry Is Related to mtDNA Methylation and Vasoconstriction in Vascular Grafts

**Figure 5:**
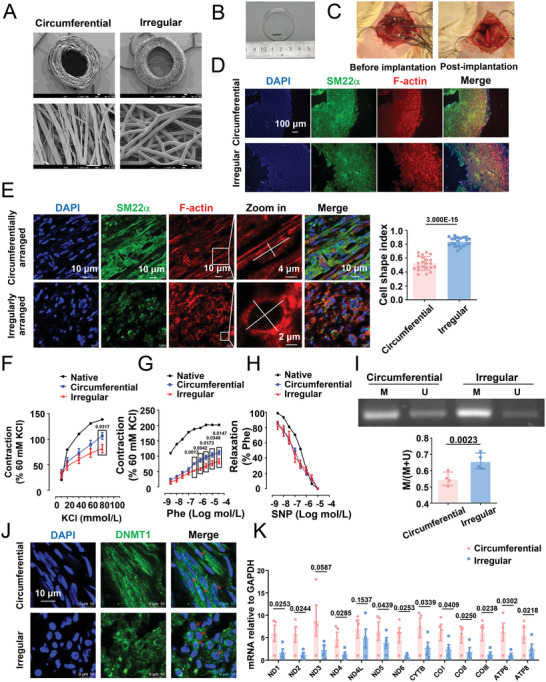
In circumferentially/irregularly arranged vascular grafts, the geometry of SMCs is related to mtDNA methylation or vessel contractility. A) Micrographs obtained using scanning electron microscopy (SEM) showing the structure of the cross‐section/longitudinal section of the circumferentially/irregularly arranged vascular grafts. B) A gross image of the grafts. C) Implantation surgery (before and post) in Sprague–Dawley rats. D) Representative immunofluorescence staining of the smooth muscle phenotypic marker SM22*α* at 3 months after implantation. E) Representative immunofluorescence staining of SM22*α* and F‐actin at 3 months after implantation. The calculation of the SMC cell shape index in the grafts is shown in the right panel. Each dot represents a single cell. F,G) KCl‐ and phenylephrine (Phe)‐stimulated vasoconstriction in the graft segments and native vessels. H) Sodium nitroprusside‐stimulated vasodilation in the graft segments and native vessels. In (F)–(H), data were obtained from eight animals. I) Representative agarose gel electrophoresis of the products of MSP for the D‐loop of bisulfite‐modified DNA isolated from circumferentially aligned or irregularly arranged vascular grafts. Semi‐quantification of the MSP results is presented in the right panels. M: methylated, U: unmethylated. Data were obtained from four animals. J) Representative immunofluorescence staining of DNMT1 in the grafts at 1 month after the implantation. The red arrows indicate the nuclear distribution of DNMT1 in the circumferentially aligned grafts and the cytoplasmic distribution of DNMT1 in the irregularly arranged vascular grafts. K) Quantitative RT‐PCR for assessing the expression of mtDNA genes in the grafts at 1 month after implantation. Fluorescence images were acquired using E,J) a confocal microscope or D) an epifluorescence microscope. Significance was assessed using a Student's *t*‐test. The error bars show ± SD. The exact *P* values between the indicated groups are presented.

To probe the potential effect of cell geometry on three‐dimensional growth in modulating cell function and behavior, we used previously designed vascular grafts, which were composed of circumferentially or irregularly arranged polycaprolactone (PCL) microfibers.^[^ ^41^, ^42^ ^]^ The two types of PCL grafts were characterized by different pore structures that were either elongated and circumferentially aligned^[^ ^41^ ^]^ or polygonal and randomly arranged.^[^ ^42^ ^]^ Using scanning electron microscopy, we observed structural differences in the PCL microfibrils in circumferentially/irregularly arranged vascular grafts (**Figure** **5**A). Tubular grafts (inner diameter 1.2 mm, length 1.0 cm) were implanted into the common carotid arteries of rats using a modified cuff technique^[^ ^43^ ^]^ (Figure 5B). The patency of the grafts post‐implantation was visualized by observing the blood flow through the grafts (Figure 5C). After the indicated duration, the grafts/neoarteries were harvested and assessed for cell infiltration, cell geometry, vasoactivity, and mtDNA methylation. Fluorescent imaging of the cross‐sections obtained at 3 months post‐implantation showed that the SM22*α*‐probed vascular SMCs could infiltrate the grafts (Figure 5D). F‐actin staining revealed that SMCs present in the circumferentially aligned grafts were primarily elongated, with a CSI of ≈0.5235; in contrast, cells in the irregularly arranged grafts were mostly polygonal, with a CSI of 0.8456 (Figure 5E). These phenomena support our postulation that formatting the geometry of the extracellular matrix could restrict cell spreading and eventually alter cell function and behavior. The second part of our postulation was verified based on the results of vasoconstriction and vasodilation assays in grafts/neoarteries harvested 4 weeks after implantation. KCl‐ or phenylephrine (Phe)‐induced vessel contraction was lower in neoarteries with irregularly arranged grafts than in circumferentially aligned grafts (Figure 5F,G). No significant difference was observed between the sodium nitroprusside‐induced endothelium‐independent vessel relaxation in the two neoarteries (Figure 5H). These data demonstrate that regenerated neoarteries with distinct cell geometries exhibit differential functional performances. Of note, the mtDNA methylation level in the neoarteries with circumferentially aligned grafts (mean value: 0.5502) was lower than that in neoarteries with irregularly arranged grafts (mean value: 0.6595) (Figure 5I), which supports the relationship between mitochondrial methylation and cell geometry. The immunostaining of DNMT1 and gene expression analysis of circumferentially/irregularly arranged vascular grafts isolated at 4 weeks post‐grafting indicated a cytoplasmic preference for DNMT1 localization and repression of mtDNA‐encoded genes in the irregularly arranged grafts (Figure 5J,K).

### 2.7 Cell Geometry Controls the Subcellular Distribution of DNMT1 in a PI3K/AKT‐Dependent Manner

**Figure 6:**
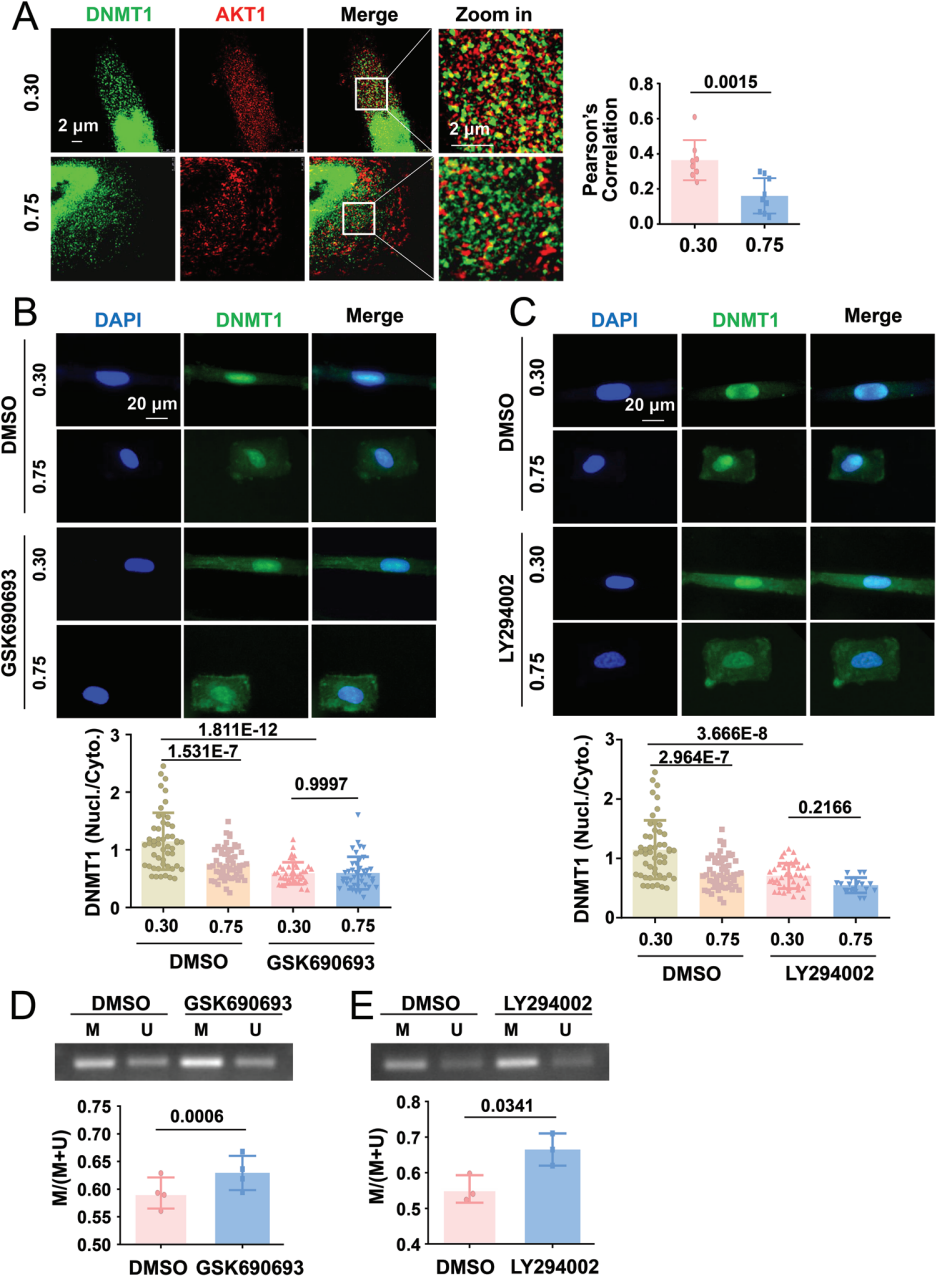
Geometric constraints control the subcellular distribution of DNMT1 in a PI3K/AKT‐dependent manner. A) Representative immunofluorescence staining and quantification of the colocalization of DNMT1 and AKT1. Images were acquired and processed using stimulated emission depletion super‐resolution microscopy. Correlation is numerically shown using the Pearson's coefficient. B,C) Micropatterned SMCs were treated with a pan‐AKT inhibitor (GSK690693, 10 µmol L^−1^), PI3K inhibitor (LY294002, 10 µmol L^−1^), or control reagent (DMSO) for 24 h; representative immunofluorescence images and quantifications of the nuclear/cytoplasmic ratio of DNMT1 are shown. Fluorescence images were acquired using an epifluorescence microscope. In (A)–(C), each dot represents a single cell. D,E) Micropatterned cells were treated with an AKT inhibitor (GSK690693), PI3K inhibitor (LY294002), or a control reagent (DMSO), and representative results of the agarose gel electrophoresis of the products of MSP for the D‐loop of bisulfite‐modified DNA isolated from the cells are shown. Semi‐quantification of the MSP results is presented in the lower panels. M: methylated, U: unmethylated. Data were obtained from four biological repeats. Significance was assessed using A,D,E) a Student's *t*‐test and B,C) two‐way ANOVA with Tukey's post hoc analysis. The error bars show ± SD. The exact *P* values between the indicated groups are presented.

In view of the cell geometry‐based regulation of DNMT1 distribution, mechanistic investigations were conducted to explore how mechanical stimuli coordinate DNMT1 localization. Others have identified phosphorylation in multiple serine and threonine residues of DNMT1, among which phosphorylation at Ser143, Ser209, and Thr249 by AKT1 was shown to stabilize DNMT1 in the nucleus.^[^ ^44^, ^45^, ^46^ ^]^ In addition, physical interactions between DNMT1 and AKT1 have been confirmed.^[^ ^44^, ^45^ ^]^ To analyze the potential regulation of DNMT1 by AKT1 in the current context, the subcellular localization of DNMT1 and AKT1 was probed in micropatterned SMCs. STED super‐resolution microscope imaging was used to detect the strong colocalization of DNMT1 and AKT1 in the cytoplasmic compartment in elongated cells and weak colocalization in square‐shaped cells (**Figure** **6**A). Analysis with a loss‐of‐function approach revealed that compared with DMSO treatment, pan‐AKT (AKT1/2/3) inhibition with GSK690693 or PI3K inhibition with LY294002 (tests for the treatment conditions are shown in Figure S14, Supporting Information) reduced the nuclear accumulation of DNMT1 in the elongated cells; GSK690693 or LY294002 eliminated the differences between DNMT1 distribution at CSI 0.30 and 0.75 (Figure 6B,C). To ensure that the observed involvement of AKT1 and PI3K had potential functional relevance, mtDNA D‐loop methylation was measured using MSP in unpatterned cells with specific treatments. As expected, both GSK690693 and LY294002 induced the hypermethylation of the mtDNA D‐loop (Figure 6D,E). Collectively, these data suggest that the subcellular distribution of DNMT1 is influenced by cell geometry, likely via the PI3K/AKT signaling pathway.

### 2.8 The Phosphorylation of AKT Target Amino Acids within DNMT1 Affects Its Responses to Geometric Constraints

**Figure 7:**
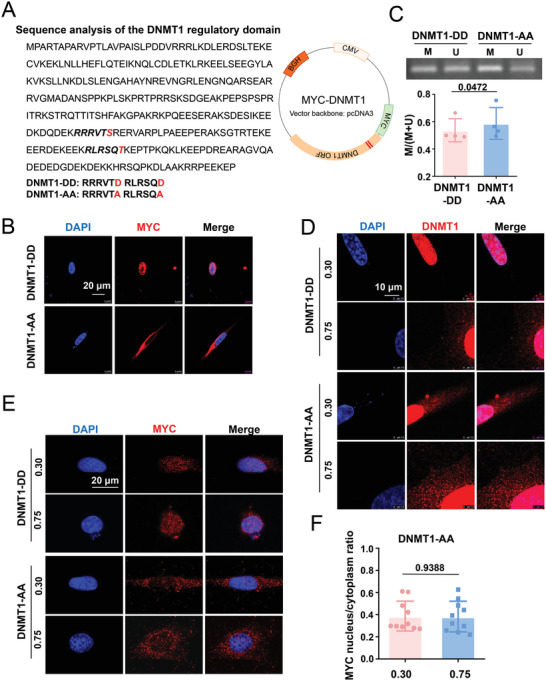
Mutagenic alteration of the AKT target amino acids within DNMT1 reinforces the cytoplasmic localization of DNMT1. A) Sequence analysis of the DNMT1 regulatory domain and construction and mutagenesis of the DNMT1 plasmids. The replacement of Ser209/Thr249 in the Myc‐DNMT1 plasmid with alanine (Ala209/Ala249, AA) generated a DNMT1‐AA mutant, whereas the replacement of Ser209/Thr249 with Asp209/Asp249 (DD) generated a DNMT1‐DD mutant. B) Representative immunofluorescence staining of Myc peptides in vascular SMCs transfected with the DD or AA plasmids. C) Representative agarose gel electrophoresis of the products of MSP for the D‐loop of bisulfite‐modified DNA isolated from SMCs transfected with the DD or AA plasmids. Data were obtained from four biological repeats. D) Representative immunofluorescence staining of DNMT1 in micropatterned cells transfected with the DD or AA plasmids. E) Representative immunofluorescence staining of DNMT1 in micropatterned cells transfected with the DD or AA plasmids. F) Quantification of the nuclear/cytoplasmic ratio of DNMT1 in (E). Each dot represents a single cell. Fluorescence images were acquired using a confocal microscope. Significance was assessed using a Student's *t*‐test (C, F). The error bars show ± SD. The exact *P* values between the indicated groups are presented.

We were unable to directly measure the phosphorylation of DNMT1 at Ser143, Ser209, or Thr249 because of the unavailability of suitable antibodies. Alternatively, mutagenesis strategies were adopted to study the critical amino acids within DNMT1 that might modulate its response to geometric stimuli. Findings from previous studies have shown that the mutagenic alteration of Ser209/Thr249 to alanine (Ala209/Ala249, AA) resulted in the loss of DNMT1 nuclear accumulation, whereas the creation of a “phospho‐mimic” amino acid (mutation to Asp209/Asp249, DD) restored its ability for nuclear compartmentation.^[^ ^46^ ^]^ We followed the design and generated the mutant DNMT1 and Myc‐fusion constructs AA and DD (**Figure** **7**A). In unpatterned vascular SMCs, the transfection of cells with AA caused the localization of exogenous DNMT1 almost exclusively to the cytoplasm, whereas transfection with DD caused the retention of DNMT1 in the nucleus (Figure 7B), which is consistent with previous findings. Accordingly, in contrast to mutation to DD, mutation to AA resulted in the hypermethylation of the D‐loop (Figure 7C), suggesting that the target amino acids of AKT within DNMT1 are indeed important for mtDNA methylation. The cells were subsequently transfected with the mutants and seeded on micropatterned substrates. Immunofluorescent staining of the cells with antibodies recognizing DNMT1 led to the detection of both exogenous and endogenous DNMT1, indicating that the DNMT1 signals in the elongated and AA‐transfected cells exhibited both nuclear and cytoplasmic localization, which was different from the nucleus‐exclusive staining observed in elongated and DD‐transfected cells (Figure 7D). The staining of the cells with the anti‐Myc antibody led to the capture of only exogenous DNMT1 and indicated a loss of geometric control in DNMT1 distribution (Figure 7E,F). These results suggest that the phosphorylation of AKT target amino acids within DNMT1 may play an important role in regulating the mechanical responses of cells.

### 2.9 cPLA2 Responds to Changes in Cell Geometry to Control DNMT1 Distribution and mtDNA Methylation

**Figure 8:**
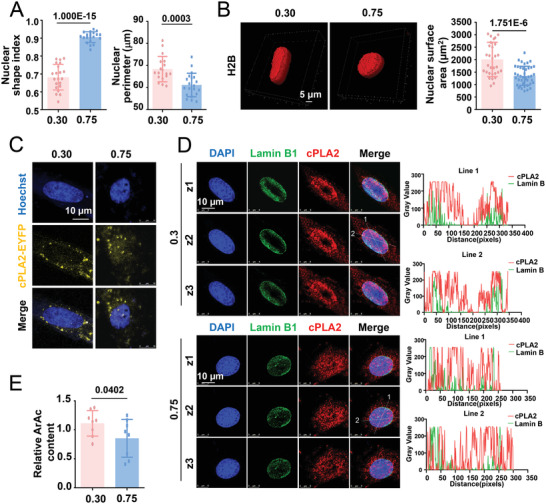
Cytosolic phospholipase A2 (cPLA2), a nuclear mechanosensor, responds to changes in cell geometry. A) The nuclear shape index and nuclear perimeter in micropatterned SMCs were calculated. B) Representative three‐dimensional reconstruction images of the nucleus of micropatterned cells and the quantification of the nuclear surface area. Cells were infected with pSIN‐H2B‐tagGFP viruses. C) Representative fluorescent images of cPLA2‐EYFP fusion proteins in micropatterned cells. Cells were transfected with the cPLA2‐EYFP plasmids before seeding. D) Representative immunofluorescent staining of cPLA2 and nuclear envelope protein lamin B1 in micropatterned cells. Z1–Z3 indicate three different scanning layers. The distribution of cPLA2 and lamin B1 and the degree of overlap between them are indicated by fluorescence intensity profiling (right panels). E) Production of arachidonic acid (ArAc) in the micropatterned cells was assayed using ELISA. Data were obtained from eight biological repeats. In (A, B) (right panel), each dot represents a single cell. In (E), data were from eight biological repeats. Significance was assessed using a Student's *t*‐test. The error bars show ± SD. The exact *P* values between the indicated groups are presented.

We attempted to understand how vascular SMCs perceive and transduce geometric signals to the PI3K/AKT pathway and downstream DNMT1 redistribution. The nucleus can detect environmental compression or stretching stimuli and respond by initiating signal transduction, thereby serving as a mechanotransducer.^[^ ^47^ ^]^ Nuclear deformation activates cPLA2,^[^ ^18^, ^19^ ^]^ which has been shown to be involved in the activation of the PI3K/AKT pathway.^[^ ^20^, ^21^ ^]^ To determine the relationship between cellular and nuclear geometries, the nuclear shape index and nuclear perimeter of the micropatterned cells were calculated; the nuclear shape indices were 0.6816 ± 0.0714 and 0. 9052 ± 0.0299 at CSIs 0.30 and 0.75, respectively. An increase in the CSI decreased the nuclear perimeter (**Figure** **8**A). To measure the nuclear surface area of micropatterned cells, fluorescently labeled histone H2B was used to display the spatial shape of the nucleus, which showed nuclear surface areas of 2009 ± 123.6 and 1352 ± 58.74 µm^2^ at CSI 0.30 versus CSI 0.75 (Figure 8B). The data suggest higher levels of nuclear deformation and membrane stretching in elongated cells than in square‐shaped cells. The observation of exogenously expressed cPLA2‐EYFP fusion proteins in micropatterned SMCs showed that they primarily formed punctate foci scattered along the nuclear membrane in elongated cells and were randomly distributed in square‐shaped cells (Figure 8C). Immunofluorescent staining followed by the scanning of the z‐axis at three different layers indicated that endogenous cPLA2 was predominantly localized to the nucleoplasm of the square‐shaped cells; however, some of the cPLA2 molecules accumulated in the nuclear membrane and colocalized with the nuclear envelope protein lamin B1 in the elongated cells (Figure 8D), which was suggestive of cPLA2 activation. Arachidonic acid content measurement in the micropatterned cells revealed that the elongated cells had a higher arachidonic acid content than the square‐shaped cells, which also suggests cPLA2 activation by cell elongation (Figure 8E).

**Figure 9:**
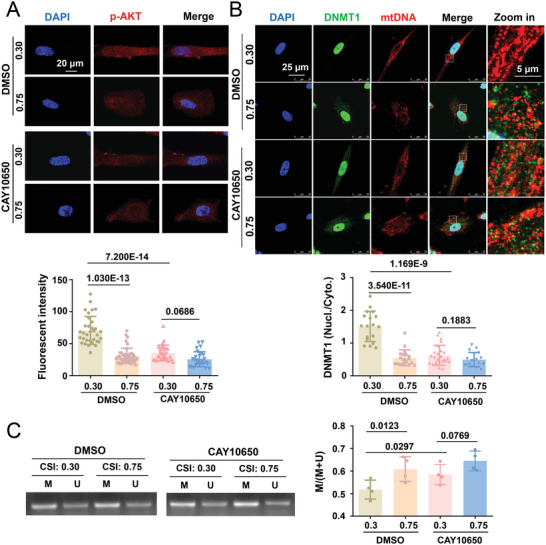
cPLA2 inhibition blocks the geometry‐regulated DNMT1 redistribution and mtDNA methylation. A) Representative immunofluorescent staining (upper panels) and quantification (lower panel) of p‐AKT in micropatterned cells treated with a cPLA2 inhibitor (CAY10650, 12 nmol L^−1^) or the control reagent (DMSO) for 1 h. B) Representative immunofluorescence staining (upper panels) and quantification (lower panel) of the nuclear/cytoplasmic ratio of DNMT1 in micropatterned cells treated with CAY10650 or DMSO. C) Representative agarose gel electrophoresis of the products of MSP for the D‐loop of bisulfite‐modified DNA isolated from micropatterned cells treated with CAY10650 or DMSO. Semi‐quantification of the MSP results is presented in the right panel. M: methylated, U: unmethylated. Data were obtained from four biological repeats. Fluorescence images were acquired using a confocal microscope. In (A, B), each dot represents a single cell. In (C), data were from four biological repeats. Significance was assessed using two‐way ANOVA with Tukey's post hoc analysis. The error bars show ± SD. The exact *P* values between the indicated groups are presented.

The activated/phosphorylated‐AKT (p‐AKT) level was higher at CSI 0.30 than at CSI 0.75 in cells treated with DMSO (**Figure** **9**A). Consistent with the in vitro findings, the p‐AKT level in the human internal mammary arteries (healthy vessels) was greater than that in the plaque areas (diseased vessels) (Figure S15, Supporting Information). Of note, the p‐AKT levels were reduced and comparable at CSIs 0.30 and 0.75 in cells treated with CAY10650, a highly potent cPLA2 inhibitor (Figure S14, Supporting Information and Figure 9A). As anticipated, the treatment of cells with CAY10650 also compromised the nuclear retention of DNMT1 in the elongated cells (Figure 9B). Moreover, CAY101650 treatment caused mtDNA hypermethylation in elongated cells to an extent similar to that observed in cells with CSI 0.75 (Figure 9C). These findings suggest that geometric signals activate cPLA2 to induce the redistribution of DNMT1.

### 2.10 Intervention of the cPLA2‐AKT‐DNMT1 Pathway Affects ATP Production and Cell Contraction

**Figure 10:**
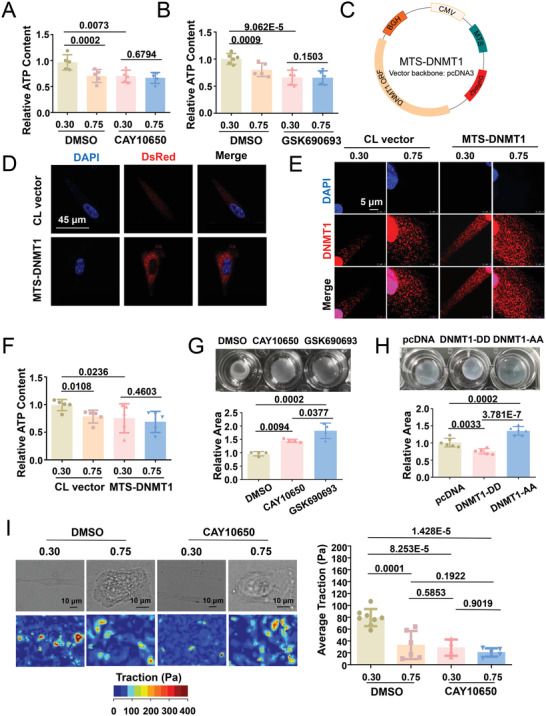
Intervention of the cPLA2‐AKT1‐DNMT1 pathway affects ATP production and cell contractility. A,B) Measurement of the intracellular ATP content of micropatterned SMCs subjected to the indicated treatments. Data were obtained from five biological repeats. C) Schematic representation of the MTS‐DNMT1 construct. MTS, mitochondrial targeting sequence, CMV cytomegalovirus promoter, ORF open reading frame. D) Representative immunofluorescent staining of *Discosoma* sp. red fluorescent protein (DsRed). Cells were transfected either with pcDNA (CL vector) or with MTS‐DNMT1. E) Representative immunofluorescence staining of DNMT1 in micropatterned cells transfected with the CL vector or MTS‐DNMT1. F) Measurement of the intracellular ATP content of micropatterned cells with pcDNA or MTS‐DNMT1 transfection. Data were obtained from five biological repeats. G) Gel contraction assay with cells with the indicated treatments. Quantifications from four biological repeats are shown in the lower panels. H) Gel contraction assay with cells subjected to DNMT1‐AA or DNMT1‐DD transfection. Quantifications from six biological repeats are shown in the lower panels. I) Traction force microscopy assay to detect the cellular contractility of micropatterned cells treated with CAY10650 (a cPLA2 inhibitor) or DMSO. Representative bright field images and cell force images are presented in the left panel. Quantification of the traction is shown in the right panel. Each dot represents a single cell. Fluorescence images were acquired using a confocal microscope. Significance was assessed using one‐way or two‐way ANOVA with Tukey's post hoc analysis. The error bars show ± SD.

To demonstrate the functional importance of the cPLA2‐AKT‐DNMT1 pathway, micropatterned cells were subjected to inhibitor treatments or transfections and assayed for ATP production. In comparison with DMSO, the AKT inhibitor CAY10650 or PI3K inhibitor GSK690693 diminished the differential regulation of ATP production in response to cell geometry and inhibited ATP production at CSI 0.30 (**Figure** **10**A,B). We used the mitochondria‐targeting sequence (MTS)‐DNMT1^[^ ^29^ ^]^ to introduce DNMT1 into the mitochondria (Figure 10C). Exogenously expressed DNMT1‐DeRed fusion proteins exhibited exclusive cytoplasmic/mitochondrial localization (Figure 10D). Immunofluorescent staining of DNMT1 in the transfected micropatterned cells confirmed the enhancement of cytoplasmic DNMT1 retention in MTS‐DNMT1‐transfected cells compared with that in control‐transfected cells at CSI 0.30 (Figure 10E). The cytoplasmic distribution of DNMT1 may impair ATP production, as ATP content measurement indicated that MTS‐DNMT1 transfection versus control transfection decreased the ATP level in the elongated cells with CSI 0.30 (Figure 10F). ATP provides energy for facilitating several processes in living cells, including cellular contraction. SMC contractility was then determined using a collagen gel contraction assay to quantify the cell‐induced contraction of the extracellular matrix.^[^ ^48^ ^]^ Compared with that in the DMSO control, the treatment of the cells with CAY10650 or GSK690693 led to the formation of larger gel areas (Figure 10G), indicating a positive contribution of the cPLA2‐AKT pathway to SMC contraction. Similar to the effect observed with cPLA2‐AKT inhibition, the transfection of the cells with DNMT1‐AA caused a marked decline in cell contraction; however, a slight increase was observed in cells transfected with DNMT1‐DD (Figure 10H). The results of the traction force microscopy assay of micropatterned cells treated with CAY10650 or DMSO indicated that the elongated DMSO‐treated cells exhibited greater traction forces than the square cells, and the treatment of the elongated cells with CAY10650 decreased the cell contractility, mimicking the effects of 0.75‐micropatterning (Figure 10I). These results revealed an important role of the cPLA2‐AKT‐DNMT1 pathway in ATP production and cell contraction.

## 3 Discussion

Mitochondrial genomes encode essential components of oxidative phosphorylation and thus play important roles in the maintenance of cellular functions. In addition to the 13 polypeptides that are solely encoded by mtDNA, the mitochondrial proteome comprises more than 1150 proteins,^[^ ^49^ ^]^ which are encoded by nuclear genes and introduced into the mitochondria through various pathways.^[^ ^50^ ^]^ Although several studies have shown that DNA methyltransferases and methylated cytosines are present in the mitochondria,^[^ ^37^, ^51^, ^52^ ^]^ which suggests the occurrence of DNA methylation in this organelle, the presence of this epigenetic modification in the mitochondria has been challenged based on contradictory findings.^[^ ^53^, ^54^ ^]^ This discrepancy might be attributed to the differences in detection techniques and cell types. In our previous study, we discovered DNMT1 and methylated cytosines in the mitochondria of PDGF‐BB‐stimulated vascular SMCs.^[^ ^29^ ^]^ We observed the differential methylation of the mtDNA D‐loop using the MSP method in SMCs with or without PDGF‐BB treatment.^[^ ^29^ ^]^ In the current study, we confirmed mtDNA methylation in vascular SMCs using HPLC‐MS and bisulfite sequencing, and our findings highlight the importance of mtDNA methylation in determining cell shape. These findings, along with the results of our previous study, demonstrate that cytosine methylation in the mitochondrial genome is likely a common epigenetic modification in vascular SMCs, and this modification could be regulated by both biochemical and biomechanical stimuli. Notably, although we observed that DNMT1 underwent a nuclear‐mitochondrial translocational shift and specifically discussed the regulation of this shift, we recognize that other DNA methyltransferases or epigenetic modifiers may also be introduced into the mitochondria under certain circumstances, as studies have indicated the presence of DNMT3A, DNMT3B, and long noncoding RNAs inside the mitochondria in support of epigenetic modifications in the mtDNA genome and the potential functional outcomes of the modifications.^[^ ^34^, ^35^, ^55^ ^]^

The molecular mechanisms by which DNMT1 is redistributed between the nucleus and mitochondria remain elusive. DNMT1 stability is regulated by various post‐translational modifications, including acetylation, deacetylation, methylation, demethylation, and phosphorylation, among others.^[^ ^56^ ^]^ As a modification for stabilization, the phosphorylation of DNMT1 increases the DNMT1 abundance in the nucleus.^[^ ^44^, ^45^ ^]^ In contrast, the dephosphorylation of DNMT1 at certain amino acid sites drastically increases the rate of nuclear‐to‐cytoplasmic transport.^[^ ^46^ ^]^ Reportedly, the enzymes catalyzing the phosphorylation of DNMT1 include AKT1, cyclin‐dependent kinase (CDK) 1, 2, and 5, and protein kinase C (PKC).^[^ ^44^, ^57^, ^58^ ^]^ AKT1 can directly interact with human DNMT1 to mediate DNMT1 phosphorylation at Ser143, Ser209, and Thr249 to stabilize nuclear DNMT1.^[^ ^44^, ^45^, ^46^ ^]^ CDKs can phosphorylate Ser154 in DNMT1 and maintain its enzyme activity and stability.^[^ ^57^ ^]^ PKC also physically associates with DNMT1 to promote the phosphorylation of DNMT1 and targets several phosphorylation sites; that said, phosphorylation at Ser127 is likely preferential.^[^ ^58^ ^]^ However, evidence to link Ser154 or Ser127 phosphorylation in DNMT1 to the subcellular distribution of DNMT1 is lacking. Owing to this, focusing on CDKs or PKC in investigations becomes challenging. Our findings showed the colocalization of DNMT1 with AKT1. More importantly, we observed that the colocalization coefficient could be significantly affected by cell shape. The findings from our loss‐of‐function studies and mutagenesis experiments indicate that Ser209 and Thr249 phosphorylation in DNMT1 via the PI3K/AKT pathway mediates mechanically regulated DNMT1 redistribution between the nucleus and mitochondria. This is physiologically relevant because the downstream functions of AKT‐mediated DNMT1 phosphorylation are likely to involve energy production and cell contraction. Further studies are warranted for the complete characterization of DNMT1 post‐translational modifications regulated in response to cell geometry and the enzymes responsible for these modifications.

The PLA2 superfamily can hydrolyze the fatty acyl group from the sn‐2 position of phospholipids with the concomitant production of lysophospholipids.^[^ ^59^ ^]^ Mammalian cells contain several structurally diverse forms of PLA2, including cPLA2, which is typically detected in the cytosol and expressed ubiquitously.^[^ ^60^ ^]^ The activation of cPLA2 was shown to be triggered by an increase in the intracellular calcium content and its phosphorylation by mitogen‐activated protein kinases, which led to cPLA2 translocation to the nuclear envelope and endoplasmic reticulum and promoted the release of arachidonic acid.^[^ ^60^ ^]^ Recent studies have confirmed that cPLA2 is a mechanosensitive protein by showing that the deformation of the nucleus can also activate cPLA2.^[^ ^18^, ^19^, ^47^ ^]^ The compression and elongation of the nucleus leads to the unfolding and stretching of the inner nuclear membrane, thus increasing the nuclear membrane tension, which triggers calcium release and activates the cPLA2‐arachidonic acid pathway.^[^ ^19^, ^47^ ^]^ The findings from these studies suggest that as a nuclear mechanoreceptor, cPLA2 induces the adaptation of cell behavior to the microenvironment when the cell undergoes deformation. Here, we also showed that cell shape restricted by micropatterns could lead to translocational responses of cPLA2 in vascular SMCs, and that cPLA2 inhibition could reduce the AKT phosphorylation and nuclear retention of DNMT1 in elongated SMCs. Our findings support the notion that cPLA2 mediates the mechanical activation of AKT and regulates DNMT1 distribution, adding to emerging evidence that supports the critical role of cPLA2 in numerous biological phenomena.^[^ ^59^ ^]^

The 13 mtDNA‐encoded proteins are essential components of the oxidative phosphorylation system and are directly involved in cellular respiration and the generation of the majority of ATP molecules to support cell metabolism.^[^ ^61^ ^]^ MtDNA damage or the insufficient expression of the encoded proteins may gradually suppress oxidative phosphorylation, ATP production, and energy‐dependent functions, such as SMC contractility.^[^ ^29^, ^30^ ^]^ In vivo evidence has shown that the SMC‐specific abrogation of the mitochondrial transcription factor gene in mice impairs arterial contractile responses to phenylephrine.^[^ ^30^ ^]^ However, the mechanism by which mtDNA expression is regulated remains unclear. The findings from our previous study showed that mtDNA transcription is regulated by PDGF‐BB‐stimulated mtDNA methylation.^[^ ^29^ ^]^ The findings presented here showed that mtDNA methylation and consequent mtDNA expression and mitochondrial functions, i.e., ATP and ROS production, mitochondrial respiration, and oxidative phosphorylation, are regulated by cell geometry, which is defined by micropatterns. One of the most interesting findings of this investigation is that cell geometry directly regulates mitochondrial gene expression and function and, in turn, SMC contractility. Furthermore, we used PCL grafts with circumferentially or irregularly arranged PCL microfibers to achieve the in vivo growth of vascular SMCs within a controlled three‐dimensional microenvironment. The methodology and findings establish an effective means of obtaining in vivo evidence of topographical effects on SMC metabolism and indicate that extracellular matrix architecture plays a critical role in determining vascular function.

## 4 Conclusions

This study reported a novel finding that geometric stimuli induce a series of intrinsically connected effects in vascular SMCs, including the subcellular redistribution of DNMT1, and alters the methylation levels of mtDNA, binding of DNMT1 to mtDNA, mtDNA gene transcription, mitochondrial function, and energy metabolism. Our study demonstrated the role of the nuclear mechanosensory protein cPLA2 and its downstream signaling molecules PI3K and AKT1 in mediating the mechanical force‐elicited cellular effects regulated by DNMT1. Our results indicate that cell geometry is a crucial determinant of SMC function; thus, the engineering of vascular grafts with controllable micro/nanostructures could be a promising strategy for improving their performance.

## 5 Experimental Section

### Cell Culture, Inhibitors, and Antibodies

Primary human umbilical artery SMCs isolated from artery of human umbilical cord were maintained in Nutrient Mixture F12 Ham Kaighn's Modification (F12K, Sigma Aldrich) supplemented with 20% (for cell maintaining) or 2% (for treatment) fetal bovine serum (FBS) (Gemini) and 10% SMC Growth Medium (Cell Applications). Cells at passage 3 to 7 were used in all of the experiments.

To inhibit cPLA2 activation, cells were incubated with CAY10650 (MedChemExpress) at 12 nmol L^−1^ for 1 h. PI3K inhibitor LY294002 (Targetmol) was used at 10 µmol L^−1^ for 24‐h‐treatment. AKT inhibitor GSK690693 (Targetmol) was used at 10 µmol L^−1^ for 24‐h‐treatment.

Rabbit polyclonal antibodies (pAbs) against DNMT1, DNMT3A, and DNMT3B were from ABclonal. Rabbit pAbs against SMA, SM22*α*, and MeCP2 were from Santa Cruz Biotechnology. Rabbit pAb against 5‐meC was from Active Motif. Rabbit pAbs against IgG and mouse mAb against ATP5a1, Lamin B1, IgG, IgM were from Proteintech. Anti‐mtDNA antibody was from PROGEN. Rabbit pAb against cPLA2, mouse mAb against AKT1, and mouse mAb against Myc‐tag were from Bioss. Mouse mAb against Dsred2 was from Origene. Rabbit pAb against phosphor‐AKT was from Cell Signaling Technology.

### Plasmids and Viruses

pcDNA3/Myc‐DNMT1, in which the full‐length human DNMT1 cDNA was cloned into EcoRI and NotI sites of pcDNA3/Myc, was from Arthur Riggs (Addgene plasmid # 36939). pDsRED2‐Mito plasmid, in which the MTS was fused to the 5′‐end of pDsRed2, was from Clontech. The MTS‐DsRED2 fragment was amplified by primer sets (forward: 5’‐TCAGAGGAGGACCTGGAATTCATGTCCGTCCTGACGCCGC‐3’ reverse: 5’‐ACCACCTGTTCCTGTAGGAATTCATGCCGGCGCGTACC‐3’) with the pDsRED2‐Mito template. The fragment was cloned into pcDNA3/Myc‐DNMT1, upstream of DNMT1 open reading frame (ORF), to generate a DNMT1 construct with mitochondrial targeting sequences mitochondrial targeting sequences (MTS‐DNMT1).

pcDNA3/Myc‐DNMT1 site‐specific mutagenesis in the regions of the two AKT binding sites was performed using PCR primers sets containing a single nucleotide difference at the point of the desired mutation.^[^ ^46^ ^]^ Primers used to mutate the serine of first putative AKT site (RRRVTSR to RRRVTAR) were (forward: 5’‐AGTTACAGCCAGAGAACGAGTTGCTAGACCGC‐3’) and (reverse: 5’‐GTTCTCTGGCTGTAACTCTACGTCTCTTCTCATCCTG‐3’); for (RRRVTSR to RRRVTDR) the primers were (forward: 5’‐GTTACAGACAGAGAACGAGTTGCTAGACCGC‐3’) and (reverse: 5’‐CGTTCTCTGTCTGTAACTCTACGTCTCTTCTCATCCTG‐3’). Primers used to mutate the threonine of the second putative AKT site (RLRSQTK to RLRSQAK) were (forward: 5’‐AGTCAAGCCAAAGAACCAACACCCAAACAGAA‐3’) and (reverse: 5’‐GGTTCTTTGGCTTGACTTCGGAGTCTCTTTTCTTCT‐3’); for (RLRSQTK to RLRSQDK) the primers were (forward: 5’‐CCGAAGTCAAGACAAAGAACCAACACCCAAACAGAA‐3’) and (reverse: 5’‐CTTTGTCTTGACTTCGGAGTCTCTTTTCTTCT‐3’).

For generation of cPLA2‐EYPF plasmid, pEnter/Flag&His‐cPLA2, in which the full‐length human cPLA2 ORF was cloned into SgfI and MluI of pEnter/Flag&His, was from WZ Biosciences. The cPLA2 fragment was amplified by primer sets (forward: 5’‐GGACTCAGATCTCGAGCTCAAATGTCATTTATAGATCCTTA‐3’; reverse: 5’‐ CATGGTGGCGACCGGTGGATCCCGTGCTTTGGGTTTACTTAGAA‐3’) with the pEnter/Flag&His‐cPLA2 template. The cPLA2 fragment was subcloned into SacI‐BamHI of pEYFP‐N1 vector to obtain the cPLA2‐EYFP plasmid.

For generation of pSIN‐H2B‐tagGFP virus, H2BC12 variant 1 was amplified from HEK 293T cDNA, and tagGFP fragment with a flexible linker at N‐terminus was amplified by PCR. Two fragments were further inserted into lentiviral transfer vector pSIN by using NEBuilder HiFi DNA Assembly Master Mix (New England Biolabs, E2621L). To package lentivirus, HEK 293T cells at ≈70% confluency were co‐transfected with pSIN‐H2B‐tagGFP, pCMV‐∆R, pCMV‐VSVG plasmids using Lipofectamine 3000 transfection reagents (Invitrogen). 2 d after transfection, viral supernatants were harvested and filtered with a 0.45 µm polyethersulfone filter.

### Cell Micropatterning

Microfabrication technique was used to create elastic membranes with micropatterns in different geometries.^[^ ^10^ ^]^ Briefly, micropatterned stamps were fabricated in PDMS by replica molding against a photoresist mold. The mold was defined on a silicon wafer using photolithography. Before coating of a SU‐8 photoresist, undesired natural oxide film on the silicon wafer surface was removed by dipping the wafer in a buffered oxide etch solution to enhance adhesion between photoresist and the silicon wafer. After spin‐coating of photoresist on the wafer, UV exposure defined the patterns by making the exposed areas chemically more stable than unexposed areas. In the coating and UV exposure process, the thickness of the coating defined the pattern height and the area of the UV exposure defined the pattern width and pitch. Photoresist developer allowed the unexposed area to remove. Before replica molding, the photoresist mold was coated with trichloro silane as an antistiction layer in a vacuum chamber for 1 h. PDMS prepolymer was mixed with a curing agent at 10:1 ratio and degassed to remove air bubbles in the mixture. To transfer the micropatterned photoresist patterns on the wafer to PDMS stamps, the mixture was then poured on the mold and thermally cured for 3 h in a convection oven at 65 °C for completer cross‐linking of PDMS. Pattern replication was completed by releasing the cured PDMS. A PDMS stamp with micropatterned was then coated with fibronectin. The cured PDMS substrates were prepared with flat surface, activated by being placed in the ultraviolet ozone cleaner at about 5 cm from the ultraviolet light source. The PDMS stamp was inverted onto the activated substrate to allow complete protein transfer. Cell suspension was added onto to the micropatterned substrate at a density of 10 000 cells cm^−2^, incubated for 6 h to allow cell adhesion.

### Immunofluorescence Staining, Microscopy Imaging, and Morphological Analysis

SMCs were fixed in 4% paraformaldehyde, permeabilized with 0.25% Triton X‐100, and were then blocked with a 5% bovine serum albumin (BSA). Cells were incubated in rhodamine‐phalloidin for 30 min to stain F‐actin. To visualize DNMT1 and other specific proteins, fixed cells were incubated with primary antibodies. After washing with phosphate‐buffered saline (PBS), the cells were incubated with the secondary antibodies. Nucleus staining was achieved with DAPI dye. Antibody specificity was validated in Figure S4 (Supporting Information). Fluorescence images were collected by a confocal microscope system (LEICA SP8) or an epifluorescence microscope (Leica DMI6000B). For STED super‐resolution microscopy (Leica TCS SP8 STED), sample acquisition was optimized using Leica SP8 software and time‐gated STED. Huygens software was used for post‐processing deconvolution. All the images were collected and subjected to morphological analysis using ImageJ. To calculate the CSI, the nuclear shape index, and nuclear perimeter, images were analyzed using Perimeter tool or Fit ellipse tool in ImageJ. The polygon selection tool was used to outline an individual cell within a given field. Geometric features (including area, grey value, perimeter, major and minor axis, circularity, aspect ratio, roundness) were automatically extracted from each cell. Cell/nuclear shape index was calculated as the equation

(1) $$\mathrm{Cell}/\mathrm{nuclear}\;\mathrm{shape}\;\mathrm{index}=\frac{4\pi S}{L^{2}}$$

where *S* is the cell/nuclear area and *L* is the cell/nuclear perimeter. To calculate the nuclear‐to‐cytoplasmic ratio of indicated proteins, images after background correction/adjustment were analyzed for fluorescence quantitative statistics. Pearson's correlation coefficient was calculated by ImageJ Coloc 2 Fiji's plugin for colocalization analysis.

For fixed tissues, OCT embedding agent (SAKURA) was used to embed tissue samples. The embedded tissues were quickly frozen and sliced. Tissue sections were washed with PBS, infiltrated with 0.25% Triton X‐100, and were blocked with 5% BSA solution. To stain SM22*α* and other specific proteins, the fixed tissues were incubated with a primary antibody, a polyclonal antibody against SM22*α*. After washing with PBS, the tissue sections were incubated with the secondary antibodies. Nucleus staining was achieved with DAPI dye. Fluorescence images were collected by a fluorescence microscope (Leica DMI6000B).

For live cell imaging, micropatterned cells infected with pSIN‐H2B‐tagGFP virus were superimposed by Z‐axis layer scan to obtain complete nuclear images, where the Z‐step size was set to be 1 µm. To calculate the nuclear surface area, the Leica Application Suite X 3D analysis software module was used to perform 3D reconstruction of the cell nucleus.

Mitochondrial morphological analysis was performed as previously described.^[^ ^62^ ^]^ The Mitochondrial Network Analysis (MiNA) toolset was used, a relatively simple pair of macros making use of existing ImageJ plug‐ins, allowing for semi‐automated analysis of mitochondrial networks in cultured mammalian cells. MiNA is freely available at https://github.com/ScienceToolkit/MiNA.

To test the specificity of the antibodies, isotype controls were employed instead of the specific primary antibodies, and the wildtype and DNMT1‐knockout mouse embryonic fibroblasts (MEFs) were used in immunofluorescence staining.

### Transient Transfection

Replacing the culture medium with OPTI‐MEM medium (Thermo Fisher) when cells grew to 70% to 80% density. Using the forward transfection method, the plasmid and Lipofectamine 2000 (Invitrogen) were mixed in proportion, incubated for 20 min, and then added directly to the medium of the cells to be transfected. Plasmid expression was detected 24–48 h after transfection.

### RNA Isolation and Quantitative Reverse Transcription (RT)‐PCR

Total RNA was extracted from cultured cells by using TRIzol reagent (Applygen) according to the manufacturer's instructions. The isolated RNA was reversed‐transcribed into complementary DNA with the M‐MLV RT system (Promega) by using Oligo (dT) primers. Real‐time PCR was performed with the 2 × RealStar power SYBR Mixture (Genestar) by using the specific primer sets. Gene expressions were normalized against GAPDH.

### Genomic DNA Isolation and MSP

For SMCs in vessels, tunica media were quick‐frozen and ground with liquid nitrogen. The cell mills were treated with 20 mg mL^−1^ proteinase K. An equal volume of Tris‐phenol solution was mixed with the sample. The mixture was centrifuged and the upper aqueous phase was transferred to a new tube. After that, an equal volume of phenol chloroform isoamyl alcohol (25:24:1) (pH>7.8) solution was added. The mixture was centrifuged, and the upper aqueous phase was transferred to a new tube. An equal volume of chloroform was mixed with the sample and centrifuged. The upper aqueous phase was transferred to a new tube. Twice the volume of ethanol and 0.2 times the volume of 10 mol L^−1^ ammonium acetate solution were then added into the sample, mixed very gently and centrifuged. The supernatant was discarded, and the resulting precipitate was genomic DNA.

Bisulfite treatment was performed with EpiMark sulfite kit (NEB). This assay entails initial modification of DNA by sodium bisulfite, converting all unmethylated, but not methylated, cytosines to uracil, and subsequent amplification with primers specific for methylated versus unmethylated DNA. The converted DNA was used as a template for MSP. MSPprimer (http://www.mspprimer.org) was used for primer design. PCR amplification was performed and the obtained PCR products were subjected to agarose gel electrophoresis. UV irradiation showed bands.

### Mitochondria Isolation and Detection of Mitochondria DNA Methylation by HPLC‐MS

Mitochondria isolation from cultured cells was performed as previously described.^[^ ^29^ ^]^ Briefly, cells were trypsinized, washed, and lysed. The cell homogenate was blended and centrifuged. The supernatant was added into a new centrifuge tube and centrifuged. The mitochondrial pellets were resuspended in appropriate incubation medium.

Detection of DNA methylation (5mC) using a HPLC‐MS method was performed as previously described.^[^ ^63^ ^]^ 1 µg of mitochondrial DNA was hydrolyzed using a DNA Degradase Plus kit (ZYMO RESEARCH). Briefly, 2 µL of DNA (500 ng µL^−1^) were mixed with 2.5 µL of 10 × DNA Degradase Reaction buffer, 1 µL of the DNA Degradase Plus nuclease mix, and 19.5 µL of sterile ultrapure water. The mixtures were incubated at 37 °C for 2 h, and were then inactivated by adding 175 µL of 0.1% formic acid. At this point, hydrolyzed DNA (5 ng µL^−1^) was obtained. Second, the hydrolyzed DNA was centrifuged in an ultrafiltration tube for DNA purification, twice at 15 000 g for 30 min each. A mixed standard of 5‐mdC and dG was prepared with a concentration of 50 ng mL^−1^. The DNA in the test samples and standards were analyzed by HPLC‐MS. A regression curve was constructed based on the mass ratio of 5‐mdC and dG substances in the mixed standard sample and the ratio of its response area in the mass spectrum, obtaining a standard working curve for quantitative genomic DNA methylation. By calculating the peak area ratio of the sample, the percentage of methylation was obtained.

### Bisulfite Sequencing

MtDNA extraction was performed in mitochondria isolated from micropatterned cells. Methylation patterns were analyzed by bisulfite sequencing conducted by iGeneTech Biotechnologies Inc., Beijing, China. In brief, extracted DNA samples were bisulfite modified using EpiTect Fast DNA Bisulfite Kit (Qiagen). The bisulfited‐treated DNA was subsequently amplified using optimized primer combinations for multiplex polymerase chain reaction. A custom Library Preparation method using the BisCap Fast Library Prep Kit created by iGeneTech was used and then library molecules were purified. Next‐generation sequencing was conducted with the Illumina HiSeq 2500 platform.

### ChIP

ChIP experiment was performed as previously described.^[^ ^64^ ^]^ In brief, cells or purified mitochondria were washed and crosslinked with 1% formaldehyde in PBS, rinsed with ice‐cold PBS, and scraped into ice‐cold PBS. The pellet was lysed and sonicated. 1 mL dilution buffer were added to the supernatant, 100 µL of mixture were used as an Input. The lysate was diluted, incubated with antibody against DNMT1 or MeCP2 for overnight at 4 °C and then with 30 µl of protein A‐G sepharose beads for another 2 h. The beads were washed, treated with RNase A (50 µg mL^−1^) and proteinase K (7.5 µL of 20 mg mL^−1^). Crosslinks were reversed at 65 °C overnight. DNA was extracted with DNA Pure‐Spin Kit (Vigorous) and subjected to PCR amplification. To verify the ChIP conditions, qPCR was performed on enriched DNA using primers against D‐loop promoter. The enrichment of immunoprecipitated DNA with anti‐DNMT1 antibody and negative control (IgG control) versus input were calculated separately with the following formula: Fold change = 2ˆ(△CT_IgG/DNMT1_ − △CT_input_).

### Detection of ATP, ROS, and Mitochondria Membrane Potential

Cells were assayed for ATP content using an ATP detection kit (Beyotime Biotechnology). The fluorescence intensity values formed by the ATP reaction in the test solution were used to determine the level of ATP content. For detection of reactive oxygen species (ROS) or mitochondria membrane potential, cells were stained with DCFH‐DA fluorescent dye (10 µmol L^−1^, Solarbio) or TMRM dye (100 nmol L^−1^, Invitrogen) for 30 min. Flow cytometry was used to detect the respective fluorescence intensity in the cells post‐staining.

### Mitochondia Respiration Assay

Mitochondia respiration experiment using Oxygraph‐2k (O2k; OROBOROS Instruments) was performed as previously described.^[^ ^29^ ^]^ Cells were digested and were then washed before being put into the electrode chambers. Substrates and inhibitors were added sequentially to determine complex I, II, and IV respiration as indicated in a previous study.^[^ ^65^ ^]^ Complex I‐supported respiration rates were measured by using 10 mmol L^−1^ glutamate +5 mmol L^−1^ malate. 5 mmol L^−1^ ADP was then added to stimulate State 3 respiration. After the addition of 1 µmol L^−1^ rotenone for the inhibition of complex I, complex II‐supported respiration was assessed with 10 mmol L^−1^ succinate. Next, Complex III‐supported respiration was inhibited by 5 mol L^−1^ antimycin, and then 0.5 mol L^−1^ N,N,N’,N’‐Tetramethyl‐p‐phenylene‐diamine (TMPD)+2 mol L^−1^ ascorbate were used to induce complex IV‐supported respiration. The intactness of the outer mitochondrial membrane was assessed by adding 10 µmol L^−1^ cytochrome C finally. The platform oxygen flux (pmol s^−1^) difference was measured by Oxygraph‐2k after every reagent was added into chambers. Cells were removed from the electrode chambers, with oxygen flux expressed as picomoles O_2_ per second per 10000 cells (pmol s^−1^/10 000 cells).

### Mitochondrial Oxidative Phosphorylation and Glycolysis

Mitochondrial metabolism was determined by measuring the OCR and ECAR of the cells with the XF‐96 Flux Analyzer by using Seahorse XF Cell Mito Stress Test kit and Glycolysis Stress Test kit (Agilent), respectively, following the manufacturer's instructions. Briefly, SMCs were seeded into Seahorse microplates and incubated at 37 °C in 5% CO_2_ for 16 h and the calibrator plate was equilibrated overnight in a non‐CO_2_ incubator. Before starting the test, cells were washed twice with assay running media (unbuffered DMEM, 25 mmol L^−1^ glucose, 1 mmol L^−1^ glutamine, 1 mmol L^−1^ sodium pyruvate for OCR; unbuffered DMEM, 1 mmol L^−1^ glutamine for ECAR) and equilibrated in a non‐CO_2_ incubator. For OCR, the analyzer plotted the value as the cells were treated by sequential injection of the following compounds: oligomycin (1 µmol L^−1^), carbonyl cyanide‐4 (trifluoromethoxy) phenylhydrazone (FCCP, 0.5 µmol L^−1^), and antimycin A (1 µmol L^−1^) plus rotenone (1 µmol L^−1^). For ECAR, the analyzer plotted the value as the cells were treated by sequential injection of the following compounds: glucose (10 mmol L^−1^), oligomycin (1 µmol L^−1^) and 2‐deoxy‐glucose (2‐DG, 100 mmol L^−1^). All readings were normalized to total protein content.

### Detection of Arachidonic acid by Enzyme‐Linked Immunosorbent Assay (ELISA)

Samples were prepared for ELISA assay in accordance with the manufacturer's instructions (Elabscience Biotechnology). In brief, the micropatterned cells were gently washed with cold PBS, trypsinized, and centrifuged at 1000 × g for 5 min to collect the cells. The collected cells were washed 3 times with cold PBS. 150 µL of PBS were added to resuspend every 10^6^ cells (protease inhibitor was added to PBS), and the cells were lysed by repeated freezing and thawing. The extracts were centrifuged at 1500 × g for 10 min at 4 °C, and the supernatants were collected for detection using the ELISA kit.

### Gel Contraction Assay

SMCs were trypsinized and resuspended in culture medium, and mixed with Collagen I solution (4 mg mL^−1^, Biocoat), 0.1 mol L^−1^ NaOH solution, F12 Ham Kaighn's Modification (2 × ) and FBS to form a gel solution, in accordance with the ratio of 1:4:1:8:2. The gel solution containing cells was added into a 24‐well plate, and were placed in a 37 °C, 5% CO_2_ cell incubator for 1 h to polymerize the gel. Culture media containing 5% FBS were added into each well. A sterile needle was used to release the gel. 6–12 h later, the degree and rate of contraction of gels as index of the contractility of the cells could be evaluated by calculating the gel surface area from acquired images. A larger gel surface indicates less cell contraction.

### Fabrication of Vascular Grafts

The circumferentially aligned grafts consisting of an external layer and an internal layer were fabricated as described previously.^[^ ^41^ ^]^ The internal layer with circumferentially orientated PCL fibers was prepared by a wet spinning technique. PCL solution (10%, w/v) in chloroform/tetrahydrofuran (3:1, v/v) was transferred into a 10 mL glass syringe equipped with a 7‐G injection needle which was submerged into a coagulation bath of oil/hexane solution (3:2, v/v). Driven by a syringe pump, the spin trickle of PCL solution was extruded into the coagulation bath at flow rate of 2 mL h^−1^. The spin trickle transformed into microsized fibers as the solvent diffuses through the rotating coagulation bath. The fibers were collected by a rotating stainless‐steel rod (1.2 mm in diameter) at 2000 rpm for 8 min. Then the fibers wrapped stainless steel rods were washed 3 times in hexane to remove excess oil and dried in a vacuum desiccator for 2 d to remove residual hexane. The fibers wrapped rod was installed in electrospinning apparatus, and the external layer with randomly arranged PCL nanofibers was prepared by the following electrospinning process. PCL solution (14%, w/v in trifluoroethanol) was loaded into a 10 mL syringe with a 21‐G needle. Electrospinning was performed under 16 kV voltage with a receiving distance of 10 cm for 3 min.

The irregular arranged scaffolds were made by electrospinning using a setup previously described.^[^ ^66^ ^]^ In brief, a polymer solution was ejected at a continuous rate using a syringe pump through a stainless‐steel needle (1.2 mm in diameter). A high voltage was applied to the needle with a variable high‐voltage power supplier. Polymer fibers were collected on a grounded rotating stainless‐steel mandrel mounted on a home‐made stand. The thicker‐fiber tubular scaffolds were fabricated with the following conditions: 25% w/v PCL in CHCl_3_/MeOH (5:1), needle‐collector distance 17 cm, flow rate 8 mL h^−1^ and voltage 11 kV with a 21‐G needle.

### Graft Implantation in Rats

All animal studies were performed in accordance with the guidelines of the Animal Care and Use Committee of Peking University and approved by the Ethics Committee of Peking University Health Science Center (LA2019102). Sprague‐Dawley rats were anesthetized and heparin (100 units kg^−1^) was administered for anticoagulation by tail vein injection. The right common carotid artery was isolated, clamped, and transected. The graft (1.2 mm in inner diameter and 1.0 cm in length) was implanted with a “cuff” technique.^[^ ^43^ ^]^ In brief, the distal side of the common carotid artery was passed through the small pieces of an 18G catheter (cuff), and then everted over the cuff and fixed with 8‐0 black silk ties. The vascular graft was then implanted between both sides of the artery and was fixed with 6‐0 black silk ties tightly enough for it not to loosen. The vascular clip was opened lightly, and it was observed that the blood flow in the vascular graft was unblocked. Postoperatively, aspirin (15 mg kg^−1^) was administered daily.

### Wire Myograph

The grafts were dissected and cleaned from connective and fat tissues. The rings of 3 mm in length were obtained and bathed in the standard Krebs buffer at 37 °C and gassed with carbogenic mixture. All preparations were stabilized under a resting tension of 1.00 g for 1 h with the buffer changed every 15 min. The presence of functional smooth muscle cells was indicated by the contractile responses induced by adding KCL (60 mmol L^−1^). After washing several times in warm Krebs buffer, KCl and phenylephrine were used to stimulate the vascular grafts, and the changes in contractile force were monitored. Stimulation with sodium nitroprusside shows the endothelial‐independent relaxation response of blood vessels. Isometric forces were recorded with force transducers connected to a PowerLab/870 Eight‐channel 100 kHz A/D converter (AD Instruments, Sydney, Australia).

### TFM

Polyacrylamide (PA) gel substrates were prepared by mixing acrylamide, bis‐acrylamide, and fluorescence beads with a diameter of 0.2 µm (Thermo Fisher), ammonium persulfate and tetramethylethylenediamine in ultrapure water. The mixture was added to glass‐bottomed dishes and then the gel surfaces were activated. Then, the micropatterned stamps coated with fibronectin were transferred to the activated gel surface and incubated for 30 min. SMCs were seeded on the gel substrates. A spatial map for each dish of fluorescent beads that were embedded within the gel substrate directly underneath the cells was taken by a fluorescence microscope (Leica DMI6000B). Following detachment of cells from the substrates using 0.5% trypsin, a second spatial map of the same beads was obtained. Monolayer displacement was calculated by comparing the two maps using a Fourier‐based difference‐with‐interpolation image analysis18. To characterize the contractile forces of each cell, the elastic strain energy stored in gels due to cell tractions was calculated as the product of local tractions and deformations, integrated over the spreading area of the cells.^[^ ^67^ ^]^

### Human Specimens

All samples were obtained with informed consent from the patients and approved by the Peking University People's Hospital Medical Ethics Committee (2015PHB024). The experiments using human specimens were carried out in accordance with the approved guidelines. Endarterectomy specimens and internal mammary arteries were obtained from patients underwent coronary artery bypass grafting or with carotid occlusive diseases. Abdominal aortic aneurysm specimens were obtained from patients underwent open surgical repair.

### SEM

Vascular grafts were immersed in liquid nitrogen for freezing. The vascular grafts were then cut along its cross‐section/longitudinal section with a sharp blade. Samples were affixed onto aluminum stubs with carbon tape, sputter‐coated with gold, and observed by SEM (JSM‐7900F, JEOL, Japan).

### TEM

Human arterial plaque and internal mammary artery were immediately fixed after sacrifice by immersing them in 2.5% glutaraldehyde in 0.1 mol L^−1^ PBS (pH 7.4) for at 4 °C. The tissues were then washed three times with 0.1 mol L^−1^ PBS solution, post‐fixed in 2% osmium tetroxide, dehydrated using a standard series of ethanol and propylene oxide concentrations, and then embedded in epoxy resin. One‐micrometer sections were stained with methylene blue, and tissues were then sectioned on an ultratome with a diamond knife. Sections were stained with uranyl acetate and lead citrate, examined and photographed using a TEM (JEM‐2000 EX, JEOL, Japan).

### Statistical Analysis

Data were expressed as Mean ± SD from at least three independent experiments. Results were analyzed by GraphPad Prism 9.0 software for statistical significance between treatment groups. For the detection of mRNA levels, HPLC/MS experiments and mitochondrial functional analyses, since each data set is the average value of a large number of cultured cells, it was assumed that the sampling distribution became a normal distribution. For cell morphological analyses and the detection of vasoconstriction function, the *n* value represents the number of single cells or animals. Normality of data distribution was tested with a D'Agostino‐Pearson or Shapiro‐Wilk test. For normally distributed data, differences between treatment groups were determined using paired or unpaired *t*‐test for two groups of data and one‐way or two‐way ANOVA for multiple groups of data. Statistical significance among multiple groups was determined by post hoc analysis (Tukey honestly significant difference test). Nonparametric tests were used when data were not normally distributed, and the Mann‐Whitney test was used to examine statistical significance. Values of *P* < 0.05 were considered statistically significant.

## Conflict of Interest

The authors declare no conflict of interest.

## Supporting information

Supporting Information

## Data Availability

The data that support the findings of this study are available from the corresponding author upon reasonable request.
