# Supplementary material for: Geometric Constraints Regulate Energy Metabolism and Cellular Contractility in Vascular Smooth Muscle Cells by Coordinating Mitochondrial DNA Methylation
Source: Adv Sci (Weinh). 2022 Sep 14;9(32):2203995. doi: 10.1002/advs.202203995 (PMC9661866; doi:10.1002/advs.202203995)
Supplement: Supplementary file 1 — Supporting Information [file ADVS-9-2203995-s001.pdf]

Supporting

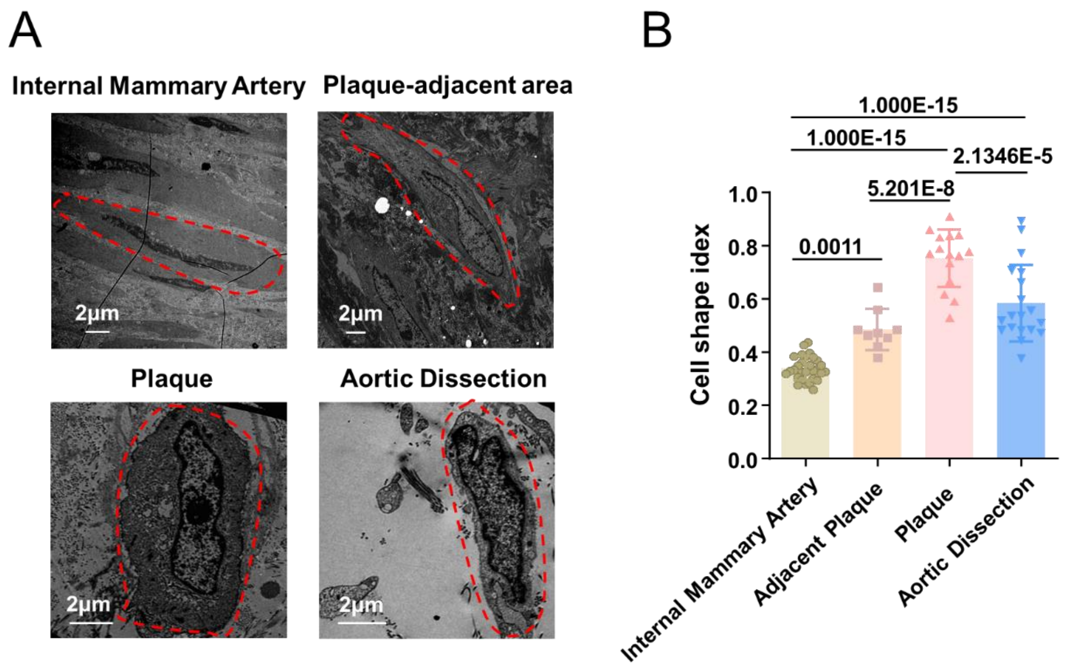

Figure S1. Geometry of vascular SMC in health and diseased blood vessels.

A Transmission electron microscopy (TEM) micrographs showing the morphology of smooth muscle cells in different areas of blood vessels. The specimens were obtained from human patients. **B** The aspect ratio (cell shape index) in A was calculated. One dot represents a single cell. Internal Mammary Artery, n= 29; Plaque-adjacent area, n=9; Plaque, n=15; Aortic Dissection, n=19. Significance was assessed by one-way ANOVA with Tukey's post hoc analysis. Error bars show ± SD. The exact *P* values between the indicated groups are presented.

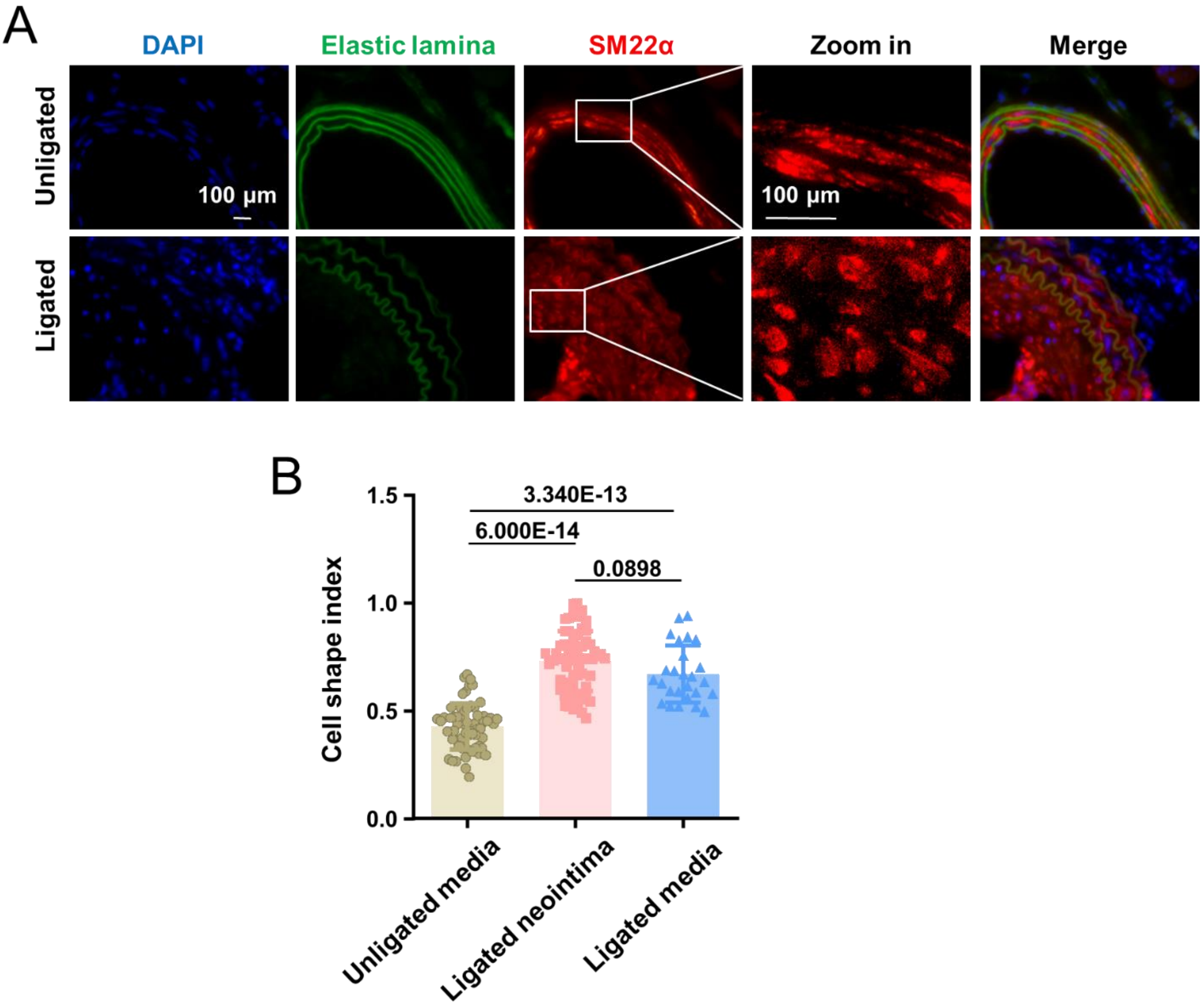

Figure S2. SMC geometry in tunica intima and media of ligated and unligated carotid arteries.

A The right common carotid artery of C57BL/6 mice was completely ligated, and the left common carotid artery was used as a control. The mice were sacrificed 30 days after ligation, and bilateral common carotid arteries were taken for immunofluorescence experiments. **B** Quantitative statistics of the cell shape index (CSI) of smooth muscle cells in control vessels and completely ligated vessels. One dot represents a single cell. Unligated media, n=61; Ligated neointima, n=61; Ligated media, n=26. Significance was assessed by one-way ANOVA with Tukey's post hoc analysis. Error bars show ± SD. The exact *P* values between the indicated groups are presented.

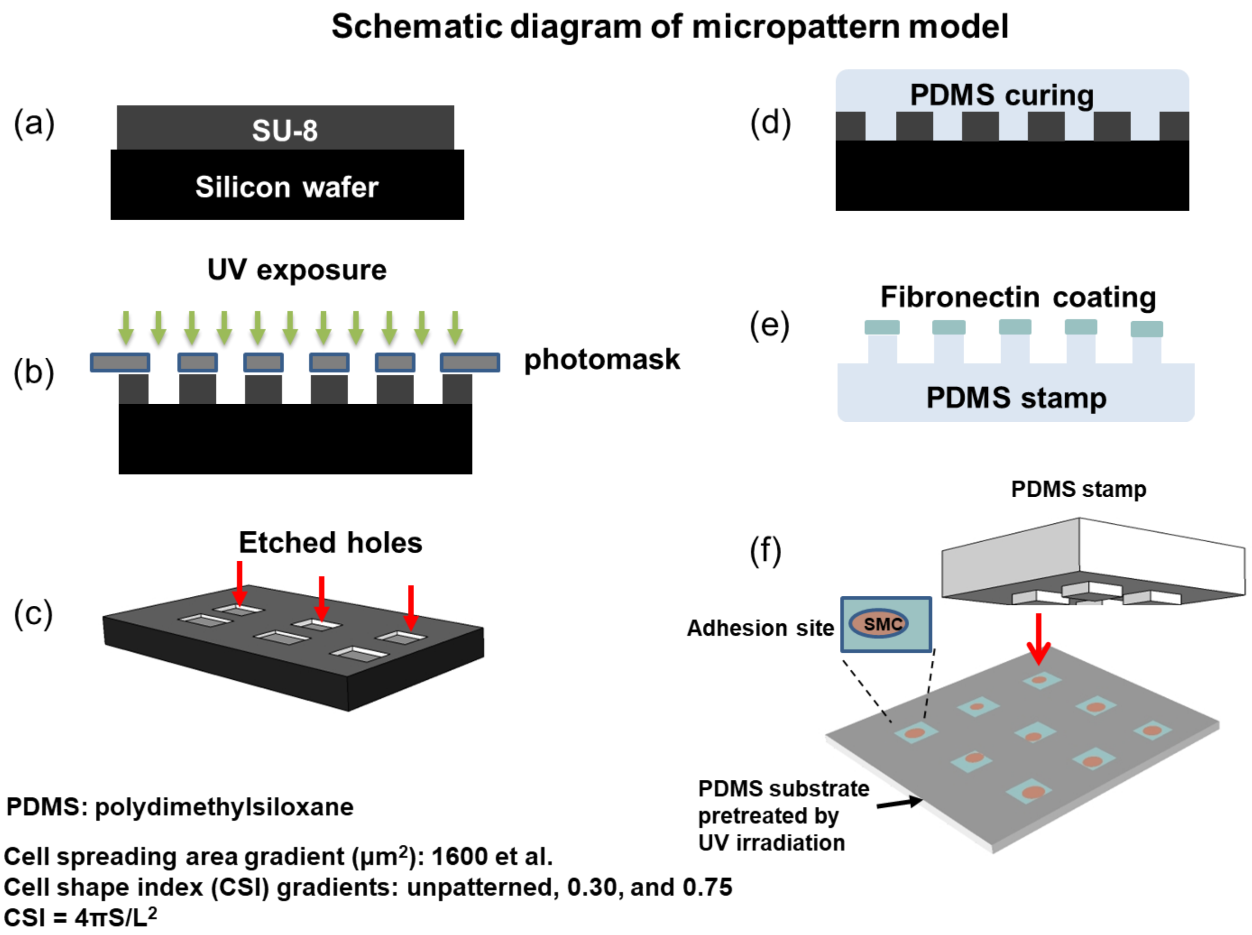

Figure S3. Manufacturing processes of the substrates with micropatterned surfaces.

Micropatterning procedures. First, the negative photoresist was spin-coated on the silicon wafer. Then, it was exposed to UV light through a photomask. Photoresist without UV-polymerization was developed away, leaving a micropatterned surface. The PDMS was cast on the silicon wafer and cured to serve as a micro-pattern mold. The fibronectin solution was incubated on the surface of the micro-patterned mold. The cells were seeded on the patterned surface and grown according to the pattern.

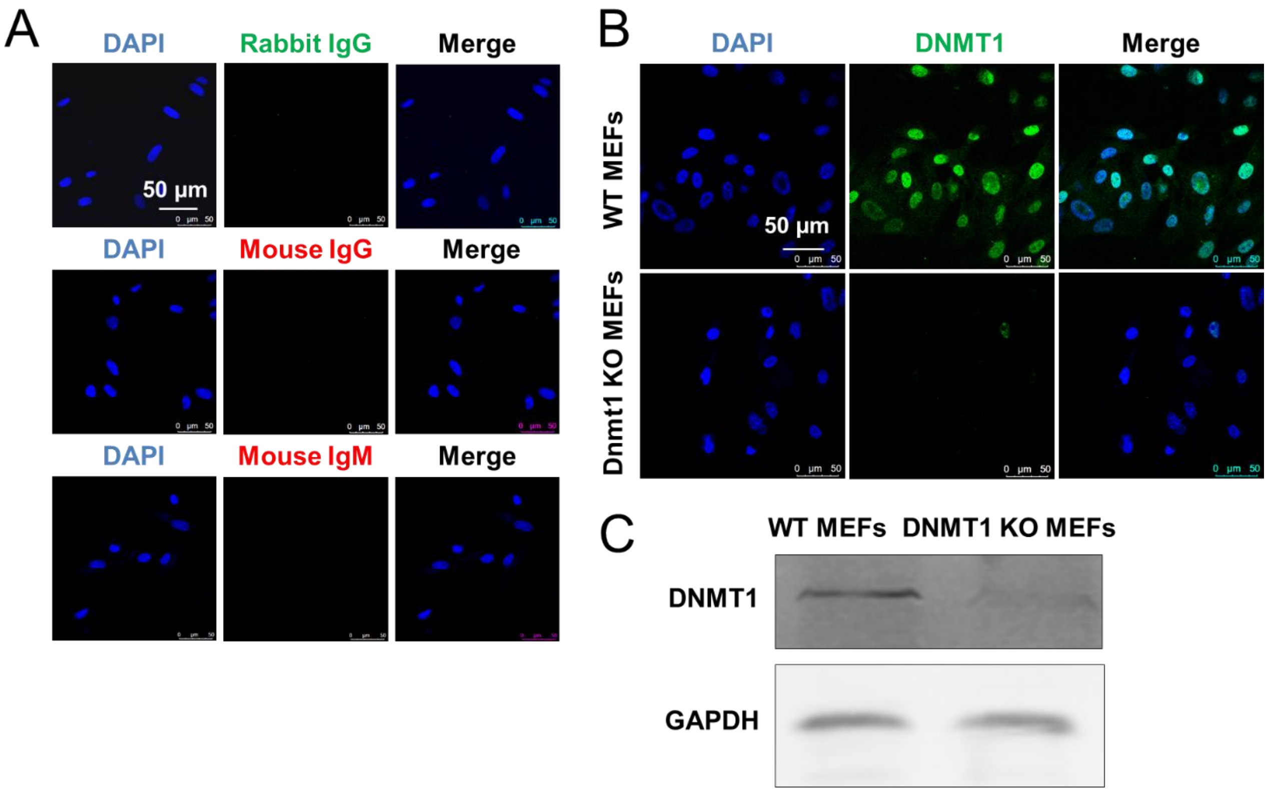

Figure S4. Validation of the antibodies.

A Representative immunofluorescence staining using the isotype controls. **B** Embryonic fibroblasts (MEFs) from DNMT1$^{flox/flox}$ mice were isolated and infected with an adenovirus expressing Cre recombinase (Ad-Cre) to specifically knockout (KO) DNMT1 expression in the cells. Shown are representative immunofluorescence staining of Dnmt1 in wild-type MEFs and DNMT1 KO MEFs. **C** Wild-type MEFs cells and DNMT1 KO MEFs cells were collected, and the expression of DNMT1 was analyzed by Western blotting.

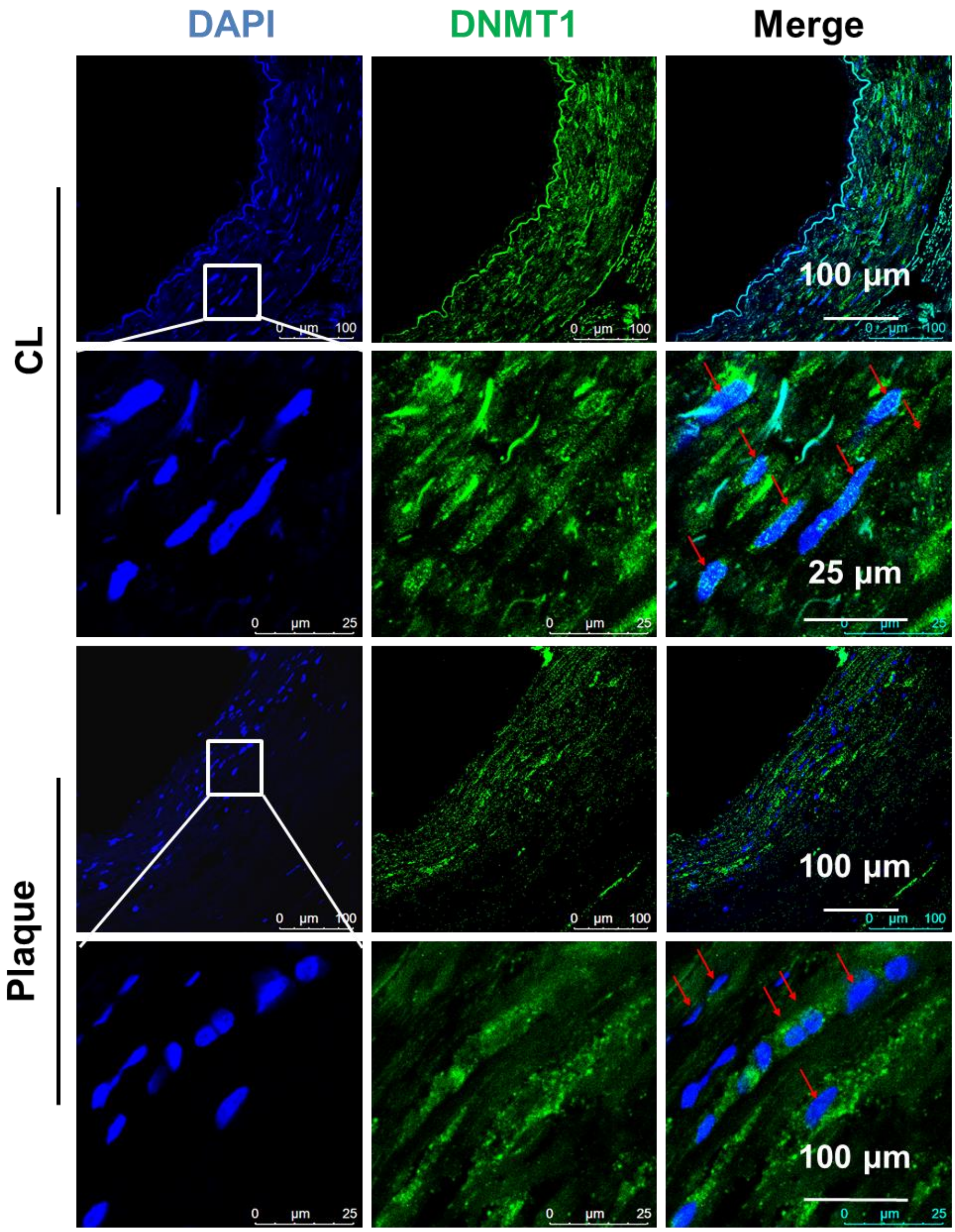

Figure S5. DNMT1 is mainly localized in the cytoplasm in vSMCs from human atherosclerotic plaque areas.

Representative immunofluorescence staining of DNMT1 in internal mammary artery (CL) and plaque area. Red arrows indicate a nuclear distribution of DNMT1 in the CL images whereas a cytoplasmic distribution in the plaque images.

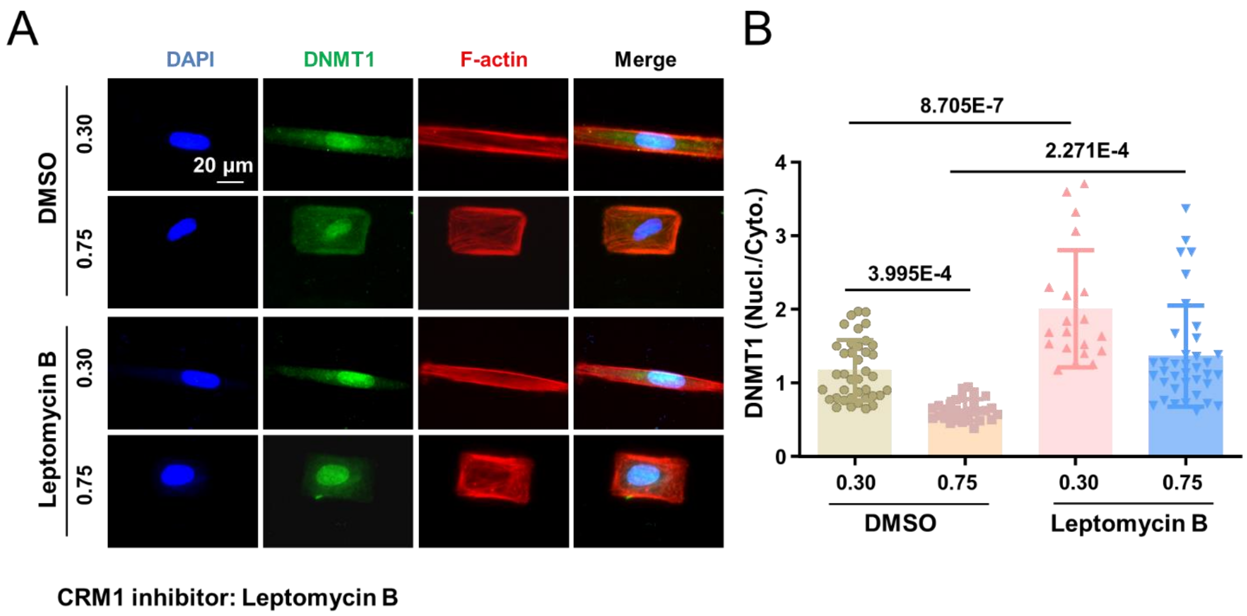

Figure S6. Leptomycin B, an Inhibitor of the nuclear export receptor CRM1, promotes a nuclear localization of DNMT1.

A After three hours of DMSO/Leptomycin B(100nM,3h) treatment of the micropatterned cells, the distribution of DNMT1 in the cells was detected by immunofluorescence staining. **B** Fluorescence quantitative statistics of DNMT1 nuclear/cytoplasmic content. One dot represents a single cell. DMSO-0.3, n=39; DMSO-0.75, n=30; Leptomycin B-0.3, n=20; Leptomycin B-0.75, n=37. Significance was assessed by two-way ANOVA with Tukey's post hoc analysis. Error bars show ± SD. The exact *P* values between the indicated groups are presented.

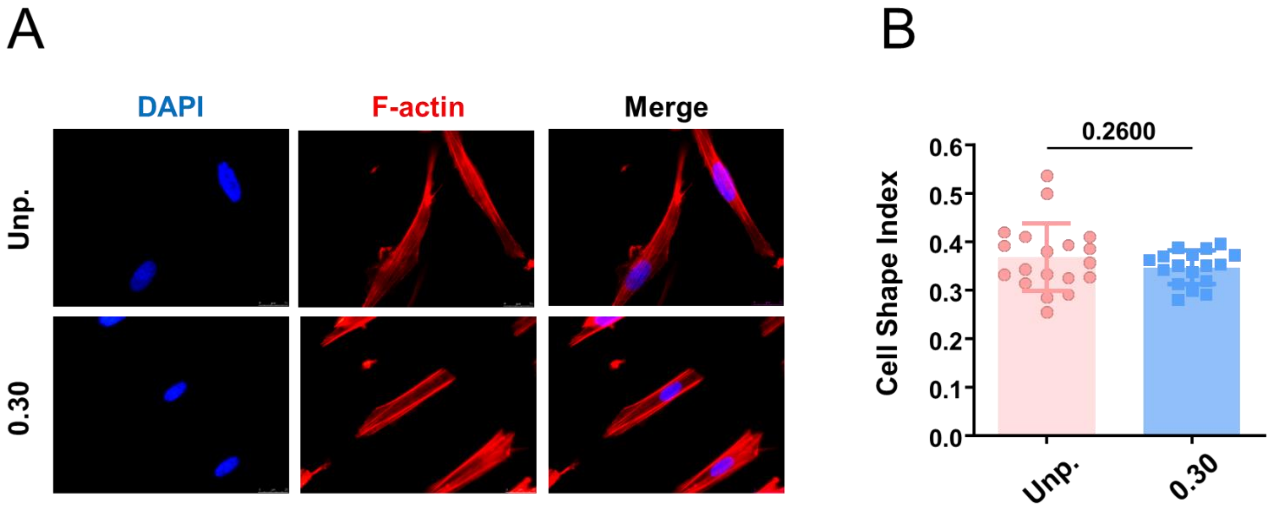

Figure S7. The morphology of SMCs in an unpatterned condition is approximately elongated.

A Representative immunofluorescence staining of F-actin in unpatterned cells and elongated cells. **B** Quantitative statistics of cell shape index. One dot represents a single cell. Unpatterned, n=19;0.30, n=17. Significance was assessed by student's t-test with Tukey's post hoc analysis. Error bars show ± SD. The exact *P* values between the indicated groups are presented.

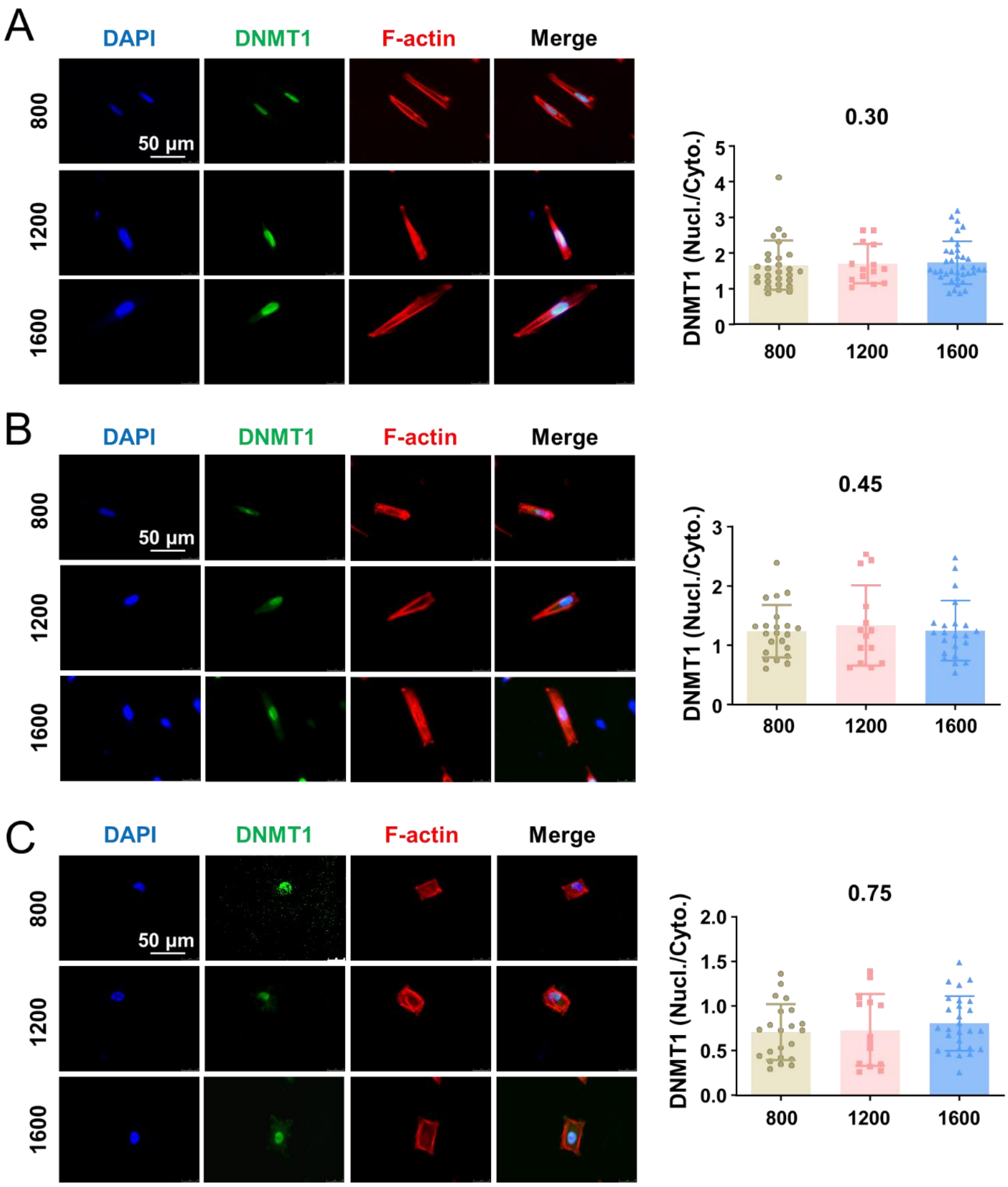

Figure S8. The DNMT1 nuclear-to-cytoplasmic ratio in cells with a fixed CSI and different spreading areas.

A SMCs were seeded on a micro-patterned surface with a CSI of 0.30 and an area of 800 μm^2, 1200 μm^2 and 1600 μm^2, respectively. After 24 hours, the cells were fixed and DNMT1 was stained.(800, n=28;1200, n=14;1600, n=37) **B** SMCs were seeded on a micro-patterned surface with a CSI of 0.45 and an area of 800 μm^2, 1200 μm^2 and 1600 μm^2, respectively. After 24 hours, the cells were fixed and DNMT1 was

stained. (800, n=22;1200, n=14;1600, n=22) **C** SMCs were seeded on a micro-patterned surface with a CSI of 0.75 and an area of 800 μm^2, 1200 μm^2 and 1600 μm^2, respectively. After 24 hours, the cells were fixed and DNMT1 was stained. (800, n=22;1200, n=15;1600, n=27) Error bars show ± SD. The exact *P* values between the indicated groups are presented.

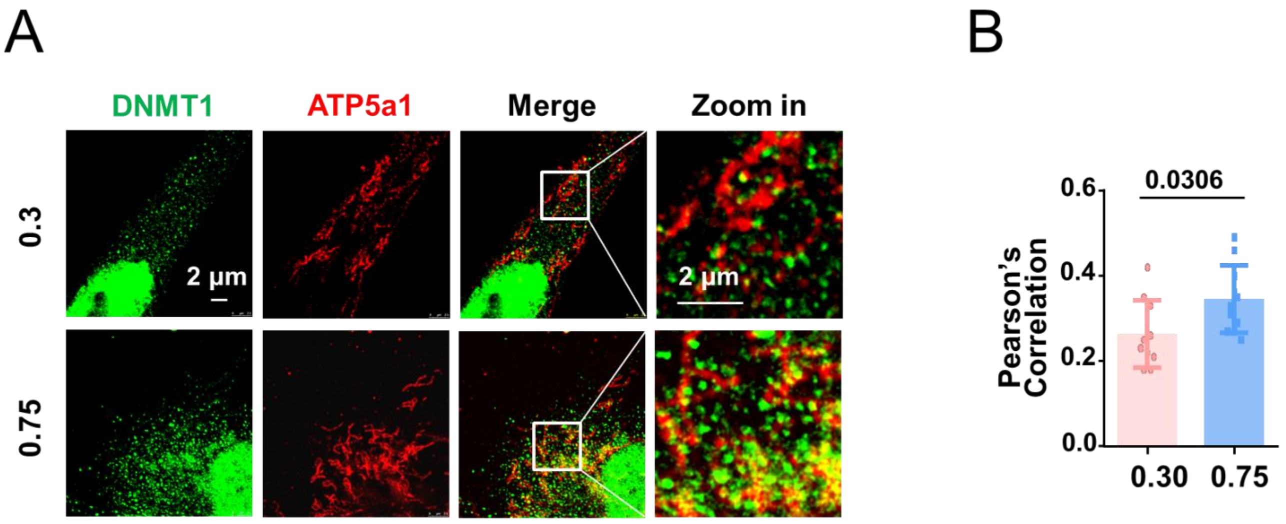

Figure S9. Higher CSI promotes co-localization of DNMT1 with the mitochondrial inner membrane protein ATP5a1.

A Representative immunofluorescence staining and quantification of the co-localization of DNMT1 and mitochondrial membrane protein, ATP5a1. Images were captured and processed by STED super-resolution microscopy. **B** The value of correlation is numerically shown by Pearson's coefficient.0.30, n=10;0.75, n=11. Significance was assessed by un-paired t-test (B). Error bars show ± SD. The exact *P* values between the indicated groups are presented.

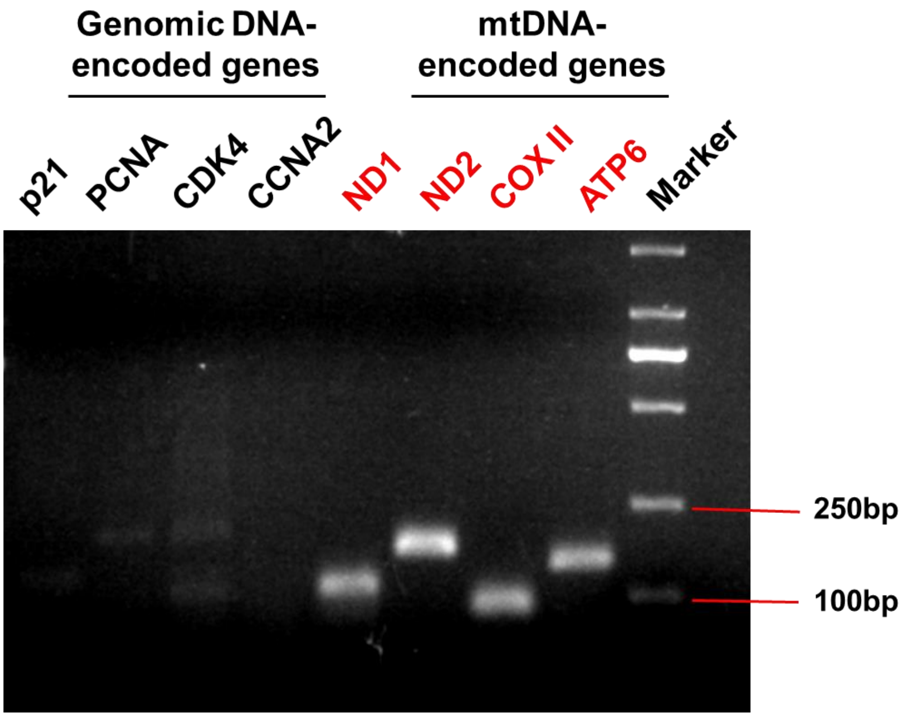

Figure S10. Validation of the purity of mitochondria isolated from SMCs.

Isolate mitochondria from smooth muscle cells were subjected to DNA extraction. PCR amplification of the extracted DNA was carried out with primers specific to genomic DNA encoding gene/mitochondrial DNA encoding gene. The purity of the PCR product was detected by DNA gel electrophoresis.

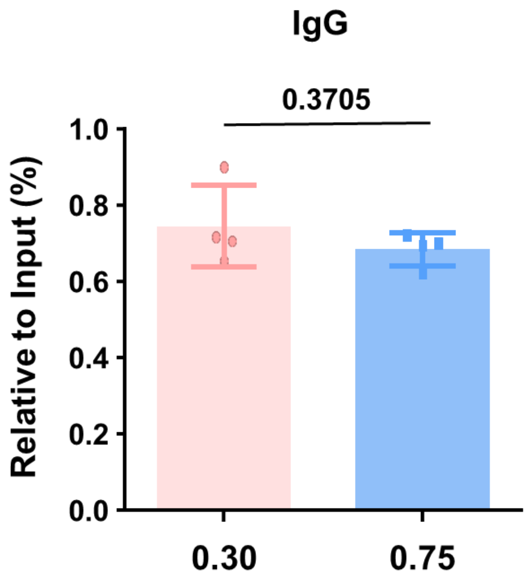

Figure S11. Chromatin IP (ChIP) assay in micropatterned SMCs using an isotype control (IgG).

The binding of IgG to the D-loop regions assessed by ChIP experiment. Data are from 4 biological repeats. Significance was assessed by student's t-test with Tukey's post hoc analysis. Error bars show ± SD. The exact *P* values between the indicated groups are presented.

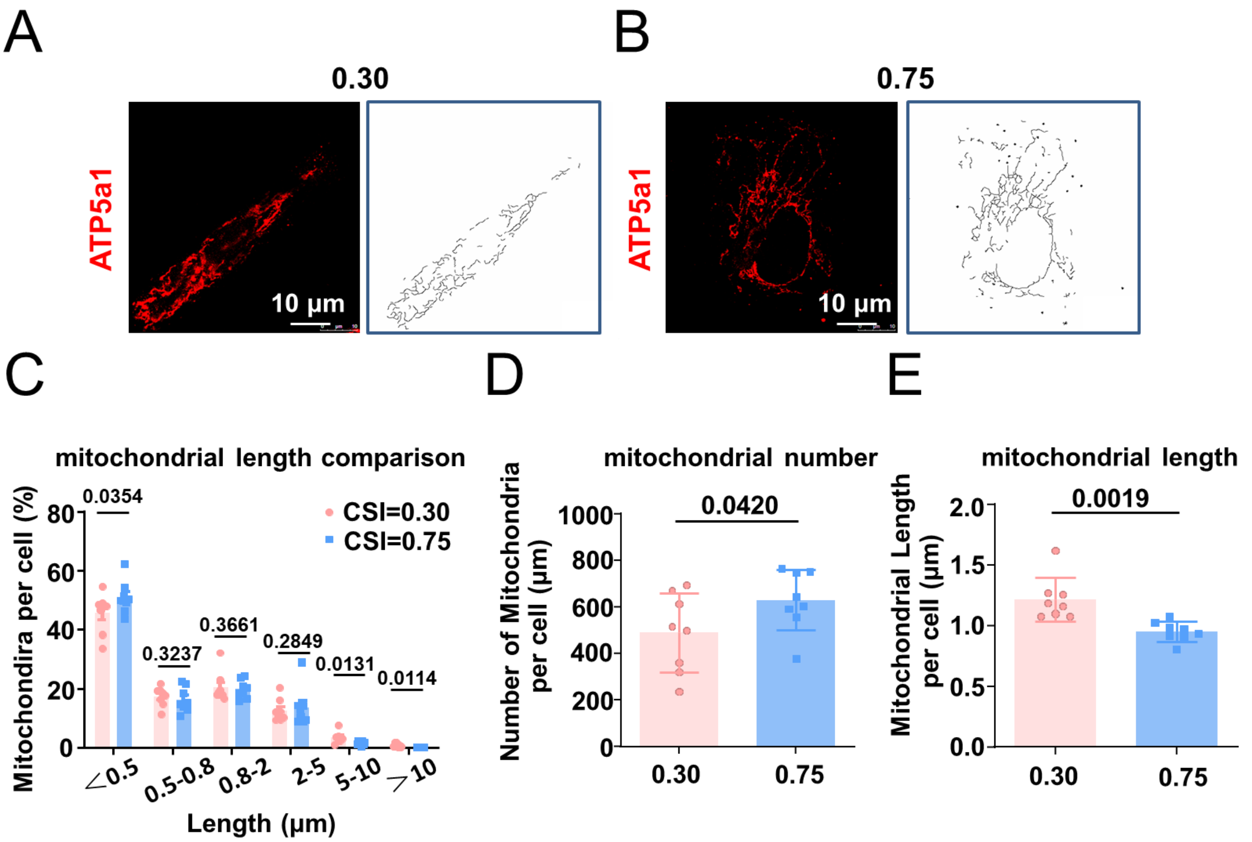

Figure S12. Analysis of mitochondrial length and number (fusion and fission) in micropatterned cells.

A Representative immunofluorescence staining of mitochondria in the elongated cells and binarized images of mitochondria. **B** Representative immunofluorescence staining of mitochondria in the square-shaped cells and binarized images of mitochondria. **C** Comparison of mitochondrial length distribution in the elongated *vs.* square-shaped cells (n=8). **D** Average number of mitochondria in the elongated *vs.* square-shaped cells (n=8). **E** Average mitochondrial length (μm) in the elongated *vs.* square-shaped cells (n=8). Significance was assessed by student's t-test with Tukey's post hoc analysis. Error bars show ± SD. The exact *P* values between the indicated groups are presented.

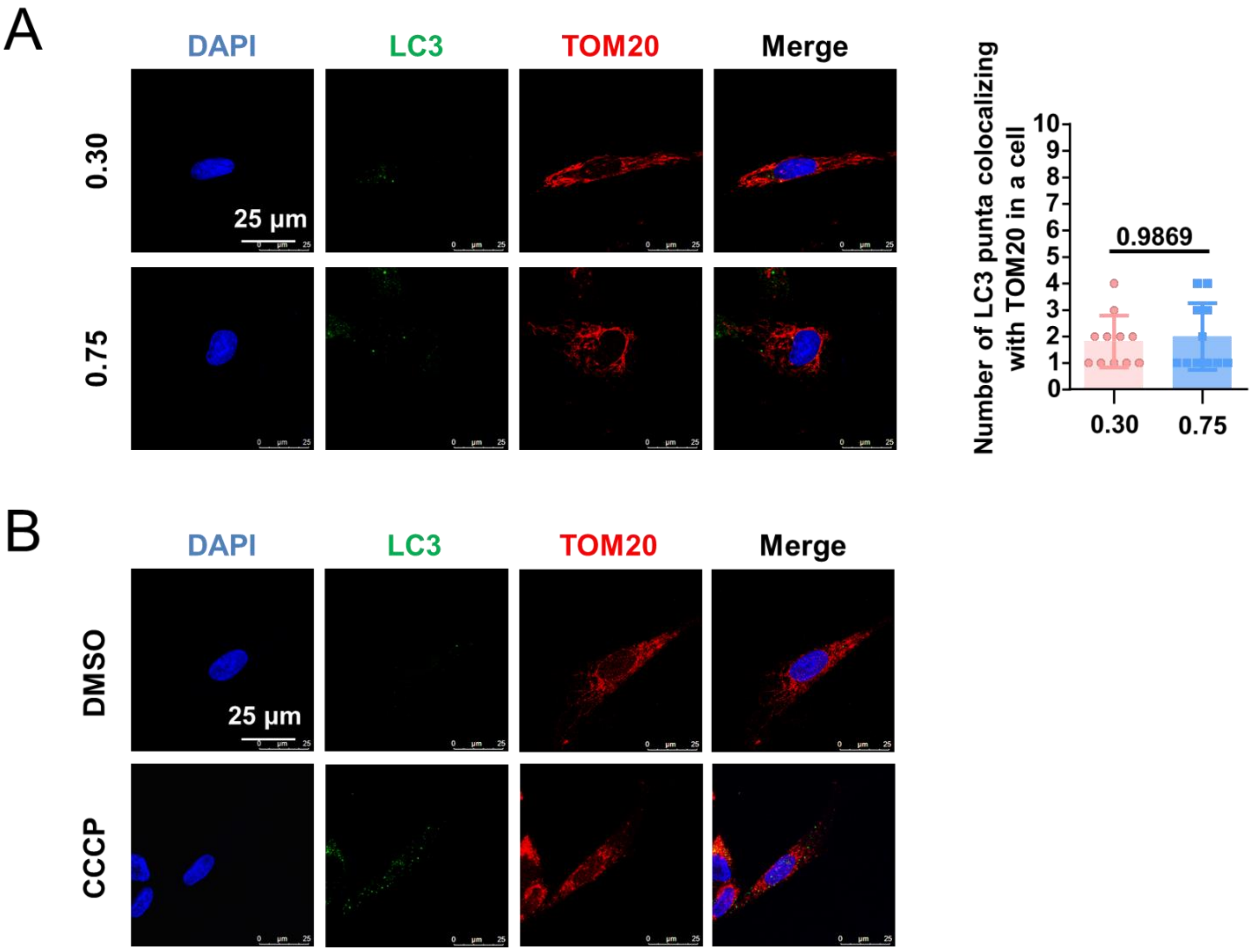

Figure S13. Analysis of mitophagy in micropatterned SMCs.

A Representative immunofluorescence staining and quantification of co-localization of autophagosomal marker LC3 with mitochondrial membrane protein TOM20 in micropatterned cells (n=11). Significance was assessed by student's t-test with Tukey's post hoc analysis. Error bars show ± SD. **B** After treatment of SMCs with DMSO or mitochondrial activator CCCP (10 μmol/L,12 hours), the co-localization of LC3 with TOM20 were detected by immunofluorescence. The exact *P* values between the indicated groups are presented.

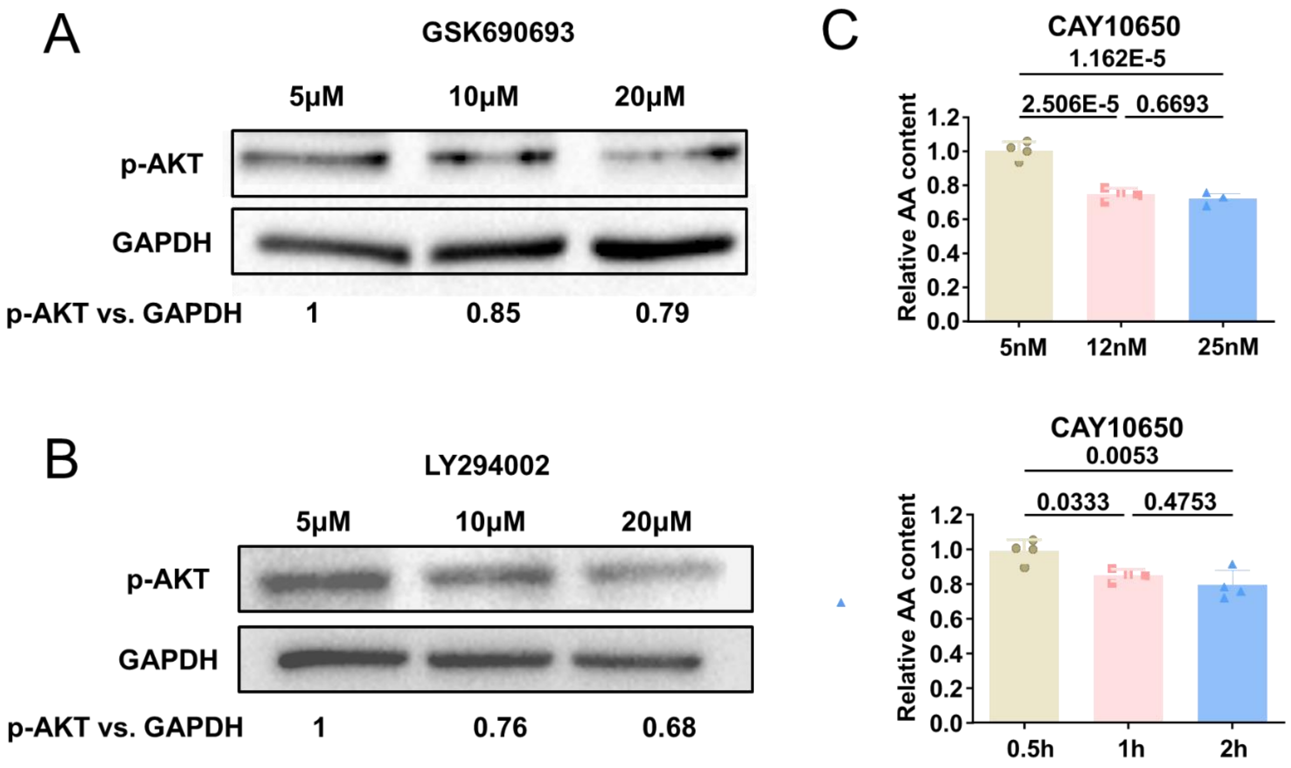

Figure S14. Validation of the inhibitory efficiency of GSK690693, LY294002, and CAY10650.

A SMCs were treated with AKT inhibitor GSK690693 at different concentrations and the expression of p-AKT was analyzed by Western blotting. **B** SMCs were treated with PI3K inhibitor LY294002 at different concentrations and the expression of p-AKT was analyzed by Western blotting. **C** SMCs were treated with different concentrations (left)/different times (right) of cPLA2 inhibitor, and the content of arachidonic acid was detected by ELISA (n=4). Significance was assessed by one-way ANOVA with Tukey's post hoc analysis. Error bars show ± SD. The exact *P* values between the indicated groups are presented.

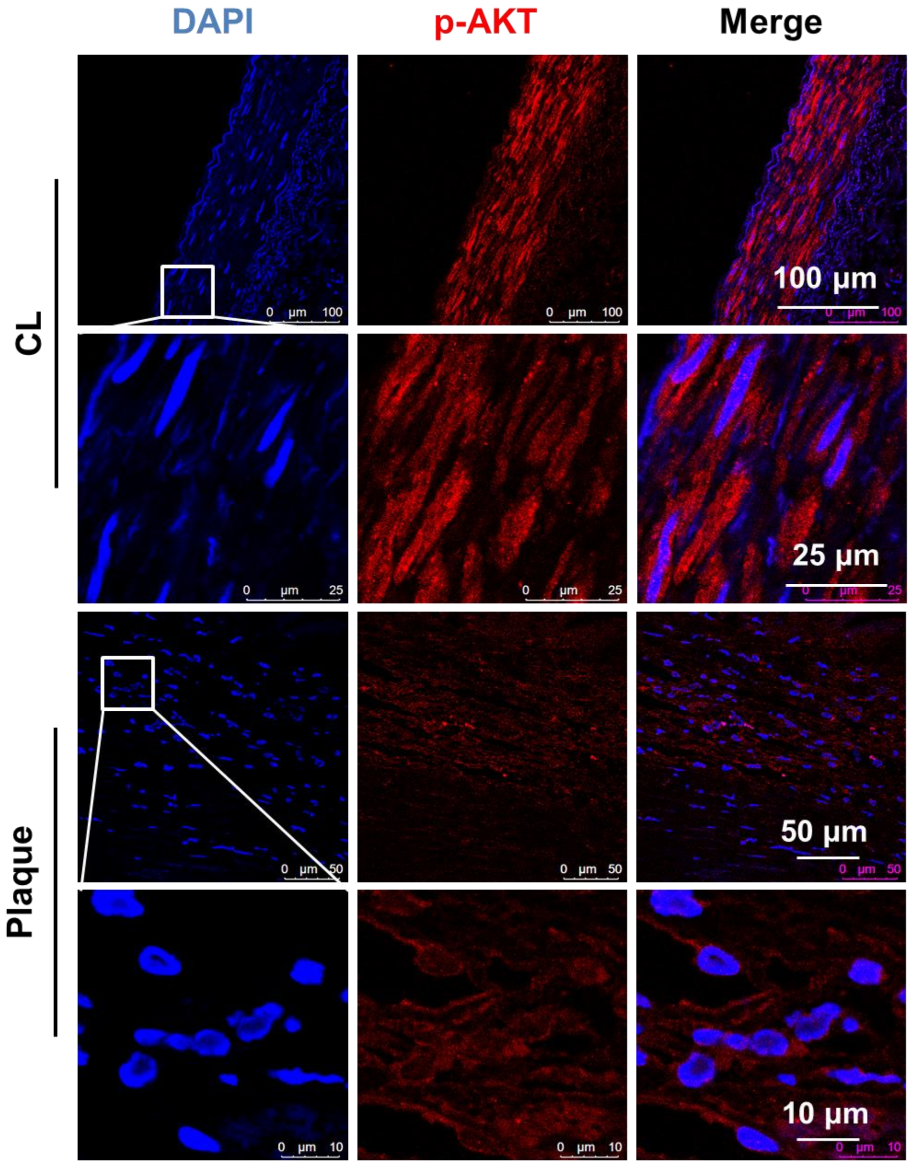

Figure S15. Expression of p-AKT in healthy and diseased vessels.

Representative immunofluorescence staining of p-AKT in internal mammary artery (CL) and the plaque area.
